# Efficient and reproducible somatic embryogenesis and micropropagation in tomato via novel structures - Rhizoid Tubers

**DOI:** 10.1371/journal.pone.0215929

**Published:** 2019-05-22

**Authors:** Wajeeha Saeed, Saadia Naseem, Daniyal Gohar, Zahid Ali

**Affiliations:** Department of Biosciences, COMSATS University Islamabad (CUI) Park Road, Islamabad, Pakistan; Nigde Omer Halisdemir University, TURKEY

## Abstract

A dual *in vitro* regeneration system consisting of indirect organogenesis and somatic embryogenesis (SE), applicable to several varieties of tomato—*Solanum lycopersicum* (cv. *Riogrande*, cv. *Roma*, hybrid *17905* and model cv. *M82*) has been established. This system is both improved and highly reproducible compared to current methods. Callus initiation, plant regeneration and SE was developed for one-week-old cotyledon explants. Indirect organogenesis via callus induction (CI) was developed for all four varieties of tomato used in this study. One-week-old tomato seedlings were used as a source of cotyledon and hypocotyl segments as explants. The explants were subsequently cultured on Murashige and Skoog (MS) medium supplemented with different combination and concentrations of plant growth regulators (PGRs). Substantial trends in regeneration and propagation response were observed among the varieties and treatments. For commercial varieties cvs. *Riogrande* and *Roma*, maximum CI was observed at 2 weeks in CIMT^9^ (0.5 mg/L NAA, 1 mg/L BAP) and CIMT^12^ (2 mg/L IAA, 2 mg/L NAA, 2 mg/L BAP, 4 mg/L KIN). However, cv. *M82* responded after 4 weeks to a combination of treatments CIMT^9^ (0.5 mg/L NAA + 1 mg/L BAP) and CIMT^13^ (2 mg/L IAA + 2 mg/L NAA + 2 mg/L BAP + 4 mg/L ZEA) for the production of calli. Subsequent shoot and root organogenesis were optimized for all four varieties. Cv. *Riogrande*, exhibited fastidious *in vitro* regeneration potential and selected for induction of somatic embryos via SE involving novel structure: rhizoid tubers (RTBs). Numerous fine hair like rhizoids (~23/explants) were first developed from cotyledon and hypocotyl explants cultured on MS medium supplemented with 0.5 or 2 mg/L NAA at pH 4.0 in dark conditions. Further incubation of each rhizoid under light conditions on MS media supplemented with 5 mg/L TDZ or BAP at pH 4.0 led to the formation of a novel structure—rhizoid Tubers (RTBs). Thus, as evident from histology, SE in *Riogrande* tomato species requires a medium with pH of (4.0) and higher concentration of cytokinins (BAP/TDZ) to form on average 40–45 RTBs from both explants. Histological and morphological studies revealed that RTBs develop through different stages of embryogenesis to multiple plantlets, on MS medium with 5 mg/L TDZ/BAP at normal pH (5.8). The results obtained indicated that the induced somatic embryos of tomato with lower pH are a more efficient mode of propagation than the organogenesis with or without callus formation. The RTBs led to a complete plantlets regeneration in 45 days compared to indirect organogenesis at 60 days.

## Introduction

Tomato (*Solanum lycopersicum*) is one of the most important edible perennial crop from the *Solanaceae* family. Relatively small genome, a diverse germplasm, and the availability of a suitable transformation system has made tomato an ideal model system for the improvement of other dicotyledonous plants [1,2]. Approximately 177 million tons of tomatoes were produced in 2016 worldwide [3]. China is playing a leading role in tomato production followed by India and the United States. Within Europe, Italy and Spain are the major producers–contributing 2/3 of the total 18 million tons of tomatoes producedin 2016 [4]. In 2014, Pakistan produced approximately 0.6 million tons of tomatoes on 62,930 hectares of land [5]. Regardless of favorable environment and available infrastructure for mass production of tomatoes, marketable yields are decreasing due to weather conditions (continuous rainy seasons and floods) and pre/post-harvest losses [6]. Being a highly perishable horticulture commodity, tomatoes are prone to post-harvest losses. These losses, however, are more prominent in developing countries where loss index can reach up to 50% annually [7,8]. Several other biotic and abiotic factors reduce the global production of tomatoes. To overcome these barriers, there has been an increased demand for the introduction of qualitative traits into commercially important cultivars of tomato in order to increase their productivity, nutritional value, and shelf life [1,9,10]. Sustainable tomato cultivation along with conventional breeding practices requires an effective regeneration system, which can exploit tissue and cell cultures for genetic amelioration with desired traits. Such system could be of potential use in somatic embryogenesis (SE), germplasm conservation and development of elite transgenic plants.

The morphogenesis and totipotency have been reported to be lower in tomatoes than that of the other members of *Solanaceae* [11,12]. In tomatoes, genotypes are sensitive, and one set of plant hormones that leads to regeneration for one species may not work for other closely related cultivars [13]. This imposes laborious constrains and prolonged regeneration responses for each cultivar. Different explant sources were used in last 4 decades for micropropagation and/or transformation in various tomato cultivars, such as leaves[14], cotyledons [15,16], hypocotyls [17], meristems[18], inflorescence [19], anthers [20] and suspension cells [21]. Many reports have indicated the process of organogenesis in tomatoes being highly influenced by factors such as genotype, origin of explant, age of explant, media composition and pH [22–24]. So far, several protocols have been developed for tomato regeneration and transformation from diverse explants and varying concentrations of plant growth regulators [11,13,25–27]. There are reports of *in vitro* recalcitrance in tomato cell cultures due to morphogenic potential, which, in turn, is largely dependent on genotypic differences. Determination of optimal conditions for cell culture impulses is a highly variable process; therefore, it can be difficult to achieve for a specific plant species or even different genotypes of the same species. Nevertheless, regeneration in tomato could be achieved via organogenesis and direct SE, where somatic embryos originated from the edges of explant (without callus formation) or indirect SE where embryos develop from dedifferentiated mass of cells (with callus formation). SE represents a complex model of totipotency regulated by cascade of signaling pathways to reprogram the fate of cell dedifferentiation and proceed to embryogenesis. Such reprogramming is often initiated by external cues such as cytokinins and auxins or stress conditions [28]. Various stress stimuli like osmotic stress, desiccation, and abscisic acid have been reported to induce the embryogenesis alone or in combination with PGRs [29,30]. Some excellent reviews have highlighted reprogramming of cellular functions in diverse plant species which are associated with stress physiology improve the regeneration potential and embryogenic response [31].

The merits of SE are versatile in biological and scientific results in clonal propagation where large number somatic embryos could be generated from single explant without intensive labor. Some potential advantages reviewed recently include SE as a substitute of direct and indirect organogenesis for *in vitro* propagation, for large scale production of true to type plants, artificial seeds and germplasm conservation and insights into gene regulatory pathways that determine fate of totipotency in plants [28,32]. The factors influencing embryogenesis response include genotypic background, explant characteristics, culture conditions such as light, pH and endogenous and exogenous PGRs [33].

Although regeneration systems for different varieties of tomatoes via organogenesis have been widely reported using explants like hypocotyls, leaf or cotyledons, proof of achieving regeneration via SE in tomatoes is limited. Few studies on somatic embryos have been completed using protoplast cultures [34], hypocotyls [35],seedling cultures [36,37] and leaf disc [38]. These studies have reported the SE in different cultivars of tomato as a quick one-step method, however without elusive generalization of established protocol was missing. This indicates that conventional protocol for achieving somatic embryogenesis in tomato is not straight forward nor widely adapted method of *in vitro* regeneration. Considering the promising results of embryogenesis in tomato, its acceptance as an application for large scale propagation and transformation of somatic embryos is an empirical process that requires more evidence [39,40]. Further, the onset of embryogenesis by stress physiology is an unexplored vista of tomato propagation. In this regard, advancements through experimentation can promote the use of SE for *in vitro* propagation of stress resilient varieties as well as genetic improvements in tomato. Consistent with this idea we used low pH with auxins as an inductive signal for embryogenesis. We attempted the development of a simple, yet robust *in vitro* regeneration protocol for different cultivars of tomato [Two commercial cultivars *cv*. *Riogrande*, *Roma*, one local hybrid-*17905* and *M82* (model cultivar)]. Various explant samples were compared and the effects of genotype, PGRs, basal media (MS) pH range and light/dark influx on somatic embryogenesis and shoot organogenesis were investigated. A new and improved regeneration system via SE by optimization of medium pH values and concentration of PGRs were developed for cv. *Riogrande*. This method of direct SE via novel structures rhizoid tubers (RTBs) is reported for the first time in *S*. *lycopersicum* cv. *Riogrande* that extends the choice of useful genotypes with a fast regeneration system that allows production of genetically engineered plants with high cytological fidelity.

## Materials and methods

### Plant material and seed disinfection

The seeds of three local tomato cultivars cv. *Riogrande*, cv. *Roma*, Hybrid -17905 (from Plant Genetic Resources, NARC-Islamabad), and a model cv. *M82* (DISAFA-UNITO-ITALY) were used in this study. Seed were stored in the dark with the temperature maintained at 4°C. Seed disinfection treatments were optimized for all varieties used in the study. Sodium hypochlorite (NaOCl) was used for sterilization by sequentially increasing the concentration from 1–20% v/v (see S1 Table). For sterilization, the seeds were first rinsed with autoclaved, double-distilled water followed by sequential immersion in 70% ethanol for 1–2 min, then in NaOCl (Phytotechnology) with or without surfactant Tween 20 (2 drops/100 ml) for 15 min. Following sterilization, the seeds were rinsed five times with deionized autoclaved water and blotted dry on sterilized filter paper. Thereafter, ten sterilized seeds were aseptically placed on glass; tissue culture jars (12 cmx10 cm) containing 30 ml of basal growth media plus vitamins (Murashige & Skoog Basal Medium with Vitamins Phytotechnology, Product No. M519) [41]. Basal medium consisted of MS salts with vitamins at a concentration of 4.15 g/L, 0.8% agar and 2% sucrose, unless stated otherwise (S2 Table). The pH of the medium was adjusted to 5.8 before autoclaving. Five tissue culture jars containing seeds subjected to different sterilization treatment in three replicates were used. The cultures were kept initially for 48 h in the dark, and subsequently maintained at 23^°^±2°C, with 30–50% humidity [42]. After the first 48 h, the cultures were switched to a 16/8 h light/dark photoperiod provided by 70 μmolm^-2^s^-1^ cool white fluorescent lights in a growth room. Germination index for the newly emerged seedling was calculated from the age of 8 days until 80% germination was achieved.

### Effect of PGRs on callus induction

Following germination, one week old, *in vitro* grown seedlings with open cotyledons were deemed suitable to be used as explants. Cotyledons of at least 2 cm were excised and both distal and proximal parts were cut to form a 0.5–1 cm explant. Hypocotyls, just below the cotyledonary node, measuring approximately 1 cm were removed from each seedling with an acropetal cut. Explants were placed horizontally with their adaxial side (cotyledons) down. The hypocotyls were also placed horizontally on a callus induction medium (CIM). Different PGRs: Naphthalene acetic acid (NAA), 6-Benzylaminopurine (BAP), Indole-3-acetic acid (IAA), Kinetin (KIN), Zeatin (ZEA), 2,4-Dichlorophenoxy acetic acid (2,4-D) and Gibberellic acid (GA3) were used independently and in combination of 17 treatments viz: CIMT^0^–CIMT^16^ **(**Table 1). For optimization, various concentrations and combinations of PGRs were tested. Different cytokinins were used in increasing concentrations while keeping a fixed, low concentration of auxins. NAA (alone), BAP + NAA, BAP + IAA, combination of two cytokinins (BAP, ZEA, KIN) and auxins analogues (NAA, IAA, 2,4-D) were tested in different concentrations. Callus induction frequency on all hormonal combinations was recorded after 4 weeks of treatment and calculated with the equation: percentage of callus induction: (No of callus forming explants/No of cultured explants) ×100.

**Table 1:** The effect of various combinations of PGRs on callogenesis in *Solanum lycopersicum* cultivars irrespective of explant type.

Treatment (Callus induction Medium) CIM	Rio Grande	Roma	Hybrid	M82
CIMT^0^ = 4.15g/L MS salts+vitamins +2%Suc+0.8% plant agar pH 5.8	0.001±0.13^h^	0.001±0.13^h^	0.001±0.13^j^	0.001±0.13^h^
CIMT^1^ = 0.2 mg/L NAA(MS basal medium)	0.001±0.13^h^	0.001±0.13^h^	0.001±0.13^j^	0.001±0.13^h^
CIMT^2^ = 0.5 mg/L NAA(MS basal medium)	0.001±0.13^h^	0.001±0.13^h^	0.001±0.13^j^	0.001±0.13^h^
CIMT^3^ = 1 mg/L NAA(MS basal medium)	0.001±0.13^h^	0.001±0.13^h^	0.001±0.13^j^	0.001±0.13^h^
CIMT^4^ = 2 mg/L NAA(MS basal medium)	1.91±0.15^g^	0.63±0.10^h^	0.01±0.97^j^	0.001±0^h^
CIMT^5^ = 0.2 mg/L NAA+1 mg/L BAP(MS basal medium)	13.7±0.04^ef^	17.83±0.042^f^	8.43±0.11^h^	0.001±0^h^
CIMT^6^ = 0.2 mg/L NAA+2 mg/L BAP(MS basal medium)	10.26±0.03^ef^	13.56±0.03^f^	10.06±0.21^h^	0.001±0^h^
CIMT^7^ = 0.2 mg/L NAA+3 mg/L BAP(MS basal medium)	2.72±0.20^g^	2.47±0.13^g^	1.26±0.1^i^	0.001±0^h^
CIMT^8^ = 0.2 mg/L NAA+4 mg/L BAP(MS basal medium)	1.5874±0.1^g^	2±0.1^g^	1±0^i^	0.0013±0^h^
CIMT^9^ = 0.5 mg/L NAA+1 mg/L BAP(MS basal medium)	82.8±0.01^ab^	75.15±0.16 ^ab^	45.64±0.12 ^ab^	30.27±0.17 ^c^
CIMT^10^ = 1 mg/L NAA+1 mg/L BAP(MS basal medium)	35.33±0.04^d^	65.40±0.06^c^	26.43±0.07^e^	4.30±0.17^fg^
CIMT^11^ = 1/0.5 mg/L IAA+1mg/LBAP(MS basal medium)	33.96±0.17^d^	28.84±0.8^e^	31.34±0.11^d^	25.45±0.09^d^
CIMT^12^ = 2 mg/LIAA+2 mg/LNAA+2 mg/LBAP+4 mg/LKIN(MS basal medium)	85.23±0.13^a^	83.93±0.11 ^a^	63.65±0.07^a^	9.86±0.51^e^
CIMT^13^ = 2 mg/L IAA+2 mg/LNAA+2 mg/L BAP+4 mg/L ZEA(MS basal medium)	46.26±0.03^c^	24.67±0.12^e^	17.5±0.21^f^	64.8±0.87^s^
CIMT^14^ = 0.5 mg/L IAA+2/0.5 mg/L NAA+2 mg/L 2,4-D+0.2 mg/L ZEA(MS basal medium)	27.05 ±0.33^d^	20.32±0.27^e^	15.32±1.2^fg^	53 ±0.67^b^
CIMT^15^ = 0.5 mg/L BAP+0.5 mg/L NAA+2 mg/L GA3(MS basal medium)	33.35±0.23^d^	35.33±0.06 ^d^	40.90±0.36^bc^	6.88±0.19^fg^
CIMT^16^ = 2 mg/L 2,4-D+0.5 mg/L BAP(MS basal medium)	14.22±0.134^e^	5.31±0.23^g^	2.0±0.16^i^	4.71±0.26^fg^

Data represent the mean ± standard error (n = 51) of three replications.

Means followed by the same letter within column are not significantly different as determined by a pairwise comparison using Tukey’s test at p<0.05.

### Effect of medium pH and auxins on rhizoids and rhizoid tubers (RTBs) formation

For the optimization of SE from cotyledon and hypocotyl explants, supplementary auxin concentrations and medium pH were tested. The effect of pH (3.0, 4.0, 5.0, 5.8, 6.0, 7.0) on the overall growth of the callus and rhizoid induction was evaluated by media formulations supplemented with increasing concentration of NAA (at 0.5, 1, 1.5, 2 and 4 mg/L) or 2,4-D (at 2, 3 and 5 mg/L). The explants were cultivated in dark conditions at 23°C ± 2°C to stimulate rhizoid formation. After a considerable number of rhizoids were formed (10–15), further sub-culturing of each rhizoid cluster was carried out on MS basal medium supplemented with 0, 5, 10, 15, 20 mg/L TDZ and/or 5 mg/ L BAP under light condition via 70 μmolm^-2^s^-1^ cool white fluorescent lights at pH 4.0. Alongside, to investigate the effect of medium pH (3.0, 4.0, 5.0, 6.0, and 7.0) rhizoid were also cultivated on optimized concentration of TDZ and BAP (5 mg/ L). The experiments were carried out in (100 mm x 15 mm) sterile plastic plates and repeated in triplicate. The number of rhizoids formed per explant and their *in vitro* morphogenesis through direct SE was recorded. Stages of RTBs formation were captured using a Nikon D5200 digital camera.

### Histochemical staining of RTBs

To study the embryonic cell masses and ontogeny of somatic embryos at different developmental stages, histochemical staining of RTBs was done. Matured RTBs that had been cultured for four weeks were fixed and dehydrated using Formalin:Glacial acetic acid:Ethanol (1:1:8 v/v/v) as a fixing solution for 24 h [43]. The samples were dehydrated using a graded ethanol series of 30%, 50%, 75%, 85%, 95% and 100% and embedded in paraffin. Samples were cut into 12 mm thick transverse sections using rotary a Microtome (Amos scientific AEM 480) and stained with 1% safranin stain (sigma). The sections were observed under an automatic scanning system (Zeiss AS3000B with Renishaw serial# 7p5015 automated imaging UK) and stereomicroscope (Olympus technologies DP 12 Japan BX41TF) and then respective images were captured at different magnifications (25–100X).

### Shoot proliferation from callus and RTBs

To optimize the direct and indirect shoot regeneration, individual explants growing calli and RTBs were inoculated on shoot induction medium (Treatment SIMT_1_–SIMT_6_). The effects of BAP (2, 3, 5 mg/L) with and without auxins (IAA, NAA) were investigated to determine the average shooting frequency and maximum number of regenerated shoots from callus. For the standard shoot induction MS salts with vitamins 4.15 g/L, 2% sucrose, 0.8% agar and pH 5.8 was used with 16 h of light incubation at 23°C±2°C. Shoot organogenesis from RTBs consisting of proembryos and cotyledonary stage embryos was initiated in two ways. First *in vitro* grown RTBs clusters were allowed to regenerate on Tuber induction medium (S2 Table). Thus, cluster of rhizoids that lead to RTBs formation at pH 4.0 were allowed for *in vivo* shoot proliferation on same medium. Secondly, *in vitro* developed RTBs were excised from explant cluster and inoculated on MS medium supplemented with increasing concentration of TDZ at pH 4.0. The effect of pH values on adventitious shoot formation with fixed concentration of TDZ (5 mg/L) was also observed in order to optimize the germination of somatic embryos emerged from rhizoids and RTBs. The explants were evaluated for average number of shoots regenerated per explant and percentage of shoot regeneration. The numbers of induced shoots were recorded after 2 weeks of incubation.

### Rooting and *ex vitro* acclimatization of adventitious shoots and RTBs

Most of the tuber shaped embryos were formed via SE from rhizoids germinated to shoots and roots on tuber induction medium (TIM). To induce root, 1–3 cm long shoots sprouting from individual calli (4 weeks) and tip of cotyledonary embryo on RTBs (2 weeks), were excised and cultivated on root induction medium (RIM) in 5.39 × 0.7 inch culture vessels with IBA (0.1, 0.2, 0.5, 1 mg/L), NAA (0.5, 1 mg/L) and IAA (0.1, 0.2 mg/L) as rooting hormone. The germinated somatic embryos with well-defined shoot tips on TIM were transferred to pH 5.8 where, they formed adventitious shoots and roots simultaneously. After 45 days, plantlets (5–6 leaf stage) roots were removed from vessels and washed to remove agar. Plantlets were transplanted to transparent plastic pots (W × D × H: 4 × 3 ×7 inches; 22 oz) containing 750 g of autoclaved potting mix (organic compost: vermiculite; 1:1 w/w) and covered with a polyethylene plastic bag (W × D × H; 8 × 4 × 12 inches, 1 MIL) with 3 holes to sustain humidity level. Plantlets were allowed to grow for 6 weeks under 23±2°C, 30–50% humidity and a 16/8 h Light/Dark photoperiod. Each plantlet was given 1ml of half strength MS with vitamins twice in a week. Moreover, on a daily basis, they were exposed to an open-air environment for hardening before being fully transferred to a glass house. Well-developed plantlets were transferred to a 900 g of substrate composed of soil:peat:organic compost 1:1:1 (w/w/w) in plastic pots (W × D × H, 4.72×3.9×5.9 inches).

### Statistical analysis

All the experiments were repeated three times with 30 explants per treatment. Significance of differences between results was estimated by one-way analysis of variance (ANOVA) using a generalized linear model. The percentage data was arcsine transformed (arcsine [squareroot (X)]) and square root (for count) before analysis. The results were back transformed and presented as mean± standard error. Variation among treatment means were compared by Tukey’s procedure at P≤0.05 using SPSS v. 23.0 (IBM, USA).

## Results

In the present study, various physical and chemical factors were optimized for somatic embryogenesis and *in vitro* regeneration of commercially important and model tomato cultivar(s).

### Seed germination and contamination control

The containment of various types of fungal or bacterial contamination during *in vitro* micropropagation is one of the major prerequisites of successful tissue culture. Seed sterilization and storage conditions affect the overall process of *in vitro* regeneration. Unfavorable conditions may lead to decreased rate and final percentage of seed germination in *Solanum lycopersicum* L. Genetic variation exists within *Solanum* species for rapid seed germination and seedling vigor and most commercial cultivars of tomato are sensitive to stress conditions during early stages of seedling growth [44].

Surface sterilization treatment for high seed vigor may result in complete loss of germination in varieties with low germination rates. In this study, the effects of different concentrations of commercial sodium hypochlorite with and without the surfactant tween 20 and household bleach on seed germination were evaluated and their efficiency to control contamination was assessed for all four cultivars of *Solanum lycopersicum* L. (see S1 Table). The addition of a 2% sucrose solution to the basal medium was tested during the germination process; it was observed that seed germination was unaffected by the inclusion or exclusion of sucrose during first week. Contamination free seed germination without sucrose inclusion in germination media was achieved in these experiments (S2 Table). Although sucrose is an important component for healthy tomato cell cultures, it was found that an absence of sucrose does not affect the rate of germination and seedling emergence (S2 Table). NaOCl has been proven to be an effective sterilant in controlling fugal and bacterial contamination [45]. An increase in concentration of NaOCl beyond 10%, v/v was found to negatively affect the seed germination and vigor. Lowest germination was observed at 15 and 20% v/v NaOCl, while ~90% seed germination was obtained at 5–6% v/v NaOCl (post 70% ethanol immersion for 1–2 min). Tween 20, a non-ionic surfactant was used as a wetting agent as it helps to penetrate aqueous NaOCl. When the 6% NaOCl solution was used alone, the germination index was lower. However, Tween 20 in combination with 6% NaOCl proved to be effective in controlling the contamination of tomato cell cultures.

### *In vitro* callus culture induction is genotype-dependent

Various PGRs control *in vitro* morphogenesis response by modulating different physiological processes. In this study, different combinations of PGRs were tested for callus induction and regeneration. The two types of explants (cotyledons and hypocotyls) from one-week-old seedlings used for callus induction showed variable responses to callus induction media treatments [CIM T^0^-T^16^] (Table 1). It was observed that the effect of explant type was of little influence as compared to genotype and treatment during the experiments. Maximum development of calli was achieved from young cotyledons of the *Riogrande* cultivar, having an efficiency of 82.08% from CIMT^9^_,_ 85% from CIMT^12^. CIMT^9^ was found to be the most suitable treatment that leading to soft, fleshy green callus that quickly regenerates. *Roma* showed callus formation on CIMT^12^ and CIMT^9^ at 83.9% and 75% respectively (Table 1).

**Fig 1:**
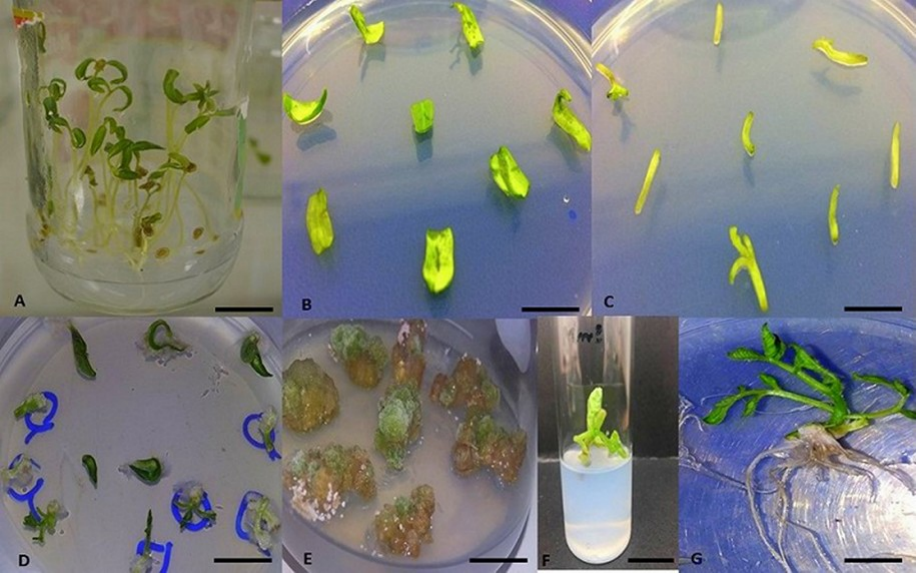
Steps of complete *in vitro* regeneration in *S*. *lycopersicum* cv. *Riogrande*. (A) *In vitro* grown one week seedlings of cv. Riogrande. (B) Cotyledonary explants. (C) Hypocotyls explants. (D-E) Calli induced from cotyledons and hypocotyls. (F-G) shoot and root organogenesis. Scale bars for (A, B, C, D, E and G), 150 mm. Scale bars for (F) 20 mm.

However, the other two genotypes i.e. hybrid (*17905*) and *M82* were comparatively less responsive at above-mentioned hormonal treatments and thus took longer time. In comparison, model cultivar *M82* only exhibited callus induction activity with a combination of treatments (CIMT^9^ & CIMT^13^/ CIMT^14^). Both CIMT^9^ and CIMT^13^ alone were found ineffective in callus formation. In order from most effective to least, different CI treatments *viz* CIMT^0^–CIMT^16^ for efficient callus induction were as follows CIMT^12^>CIMT^9^>CIMT^10^>CIMT^13^>CIMT^11^>CIMT^15^>CIMT^14^. Embryogenic calli derived from cotyledonary explants depicted a high shoot regeneration potential in *Rio* and *Roma* while other two cultivars only developed pale white compact callus (S1 Fig). A hypocotyl-derived callus had many embryoids in *Riogrande*, however, callus induction was slower, and calli were less totipotent as compared to those from cotyledons (Fig 1). The response to callogenesis was found highly dependent on genotype and was notably affected by the reproductive background mode (self-pollination) of the cultivars.

### Lower pH induced regeneration and RTBs

**Fig 2:**
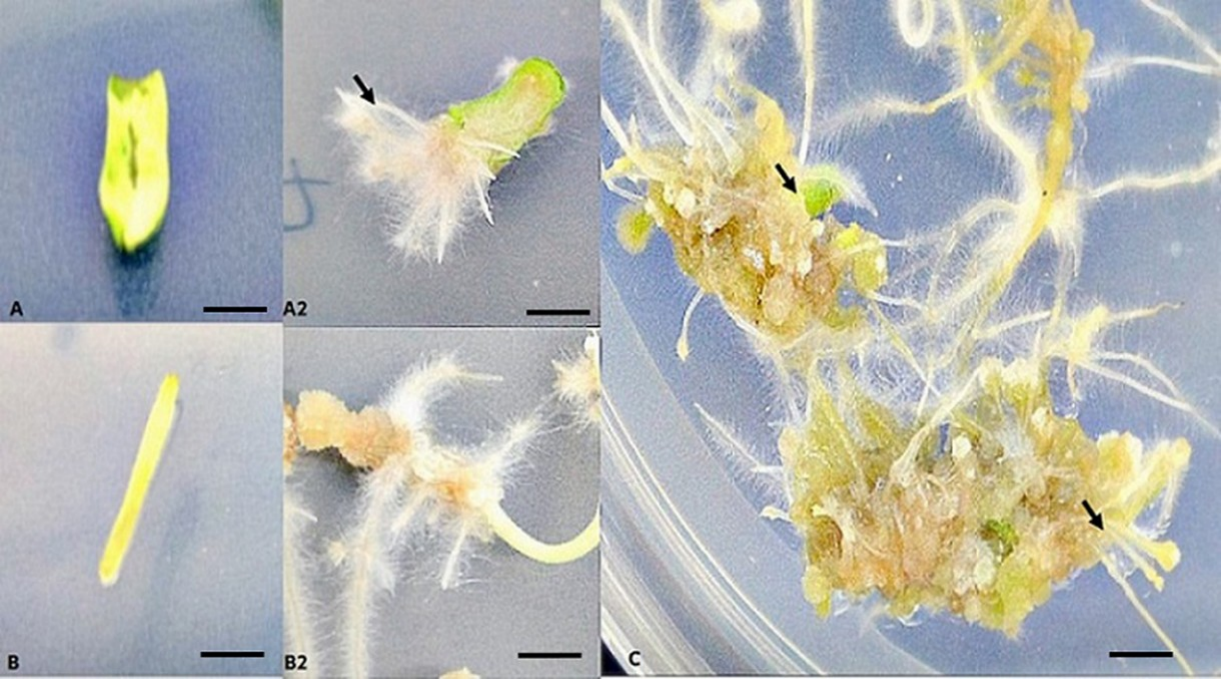
The effect of medium pH (4.0) on rhizoids production from cotyledon and hypocotyl explants of tomato (cv. *Riogrande*) with optimized concentration of NAA (0.5/2 mg/L). (A-A2) Rhizoids induced on cotyledon explants after one-week incubation. (B-B2) Rhizoids induced on hypocotyl explants after one-week incubation. (C) Primary and secondary somatic embryogenesis with many proembryos after 2 weeks of incubation. Scale bars (A, A2, B, B2) 5 mm. Scale bars (C), 50 mm.

Low medium pH (4.0) with NAA at 2 mg/L resulted in initiation of SE from one-week-old tomato cotyledons and hypocotyls irrespective of explant type. In general 2,4-D is considered as most effective hormone for induction of primary and secondary somatic embryos in many plants species [46]. Testing variable pH ranges (3–7) with two auxins NAA and 2,4-D at concentrations 0.5 mg/L and/or 2 mg/L revealed that only pH 4.0 and NAA (0.5 mg/L and 2 mg/L) favored rhizoid formation in dark condition (Fig 2A2, 2B2 and 2C) from both type of explants. Without PGRs or dark conditions, no rhizoids were observed (S2 and S3 Figs). Moreover, pH 3.0 failed to solidify to support tubers formation or morphogenesis. Different concentrations of both auxins analogues with all pH levels tested in triplicate revealed that 2,4-D was not as effective as NAA [0.5 and 2 mg/L] (Table 2). In fact, explants sub-cultured on a medium supplemented with 2,4-D failed to produce rhizoids.

**Table 2:** The effect of various concentrations of NAA and pH values on rhizoid induction in *S*. *lycopersicum* cv. *Riogrande*.

NAA (mg/L)	pH level	Mean No. of rhizoids/ explant ± S.E	2,4-D (mg/L)	pH level	Mean No. of rhizoids/ explant ± S.E	NAA (mg/L)	pH level	Mean No. of rhizoids/ explant ± S.E
0	4	00.00±0.00^d^	0	4	0.00±0.00	2	3	00.00±0.00^d^
0.5	4	23.6±3.56^a^	0.5	4	0.00±0.00	2	4	23.0±2.79^a^
1	4	15.21±2.34 ^ab^	1	4	0.00±0.00	2	5	16.31±2.11^ab^
1.5	4	8.23±1.59^bc^	1.5	4	0.00±0.00	2	6	14.72±2.22^c^
2	4	21.69±3.4^a^	2	4	0.00±0.00	2	7	11.03±1.39^c^
3	4	11.31±1.74^b^	3	4	0.00±0.00	—	—	—
4	4	9.80±1.43^bc^	4	4	0.00±0.00	—	—	—

Data represent the mean ± standard error (n = 40) of four replications.

Means followed by the same letter within column are not significantly different as determined by a pairwise comparison using Tukey’s test at p<0.05.

Nonetheless, role of auxins for induction of embryogenesis particularly 2,4-D as a stress inducer to change the fate of cell differentiation for embryo formation is well documented [47,48]. SE could be induced by use of combinations of auxins and/or cytokinins that influence endogenous levels of auxins and polar auxins transport. However, this process is largely species dependent [47]. Several auxins analogs have been alternatively used for induction of somatic embryos [49–51]. Surprisingly, a rise in concentration of NAA>2 mg/L led to callogenesis, but no significant rhizoids formation occurred at even at pH 4.0. However, NAA at a concentration 0.5 or 2 mg/L with pH 4.0 incubated at 23±2°C in dark conditions resulted in maximum number of adventitious roots as white rhizoids surrounded by many hairy root-like structures (Fig 2). Both cotyledons and hypocotyl explants displayed significant growth of rhizoids; however, the former took less time for initiation (S2 Fig). After 3 weeks of inoculation, the average number of rhizoids induced from cotyledons at pH 4.0 were 23.6±3.56 in a medium supplemented with 0.5 mg/L and 2 mg/L NAA. Testing various levels of pH against range of concentrations of NAA (0.5 mg/L−4.0 mg/L) clearly showed that only at certain threshold concertation of NAA at pH 4.0 can effectively induce SE (Fig 2). The sequence of effective media pH to exhibit a substantial number of rhizoids and RTBs from both explants was 4.0 > 5.0 > 6.0 > 7.0 supplemented with NAA 0.5 or 2 mg/L (Table 2).

**Fig 3:**
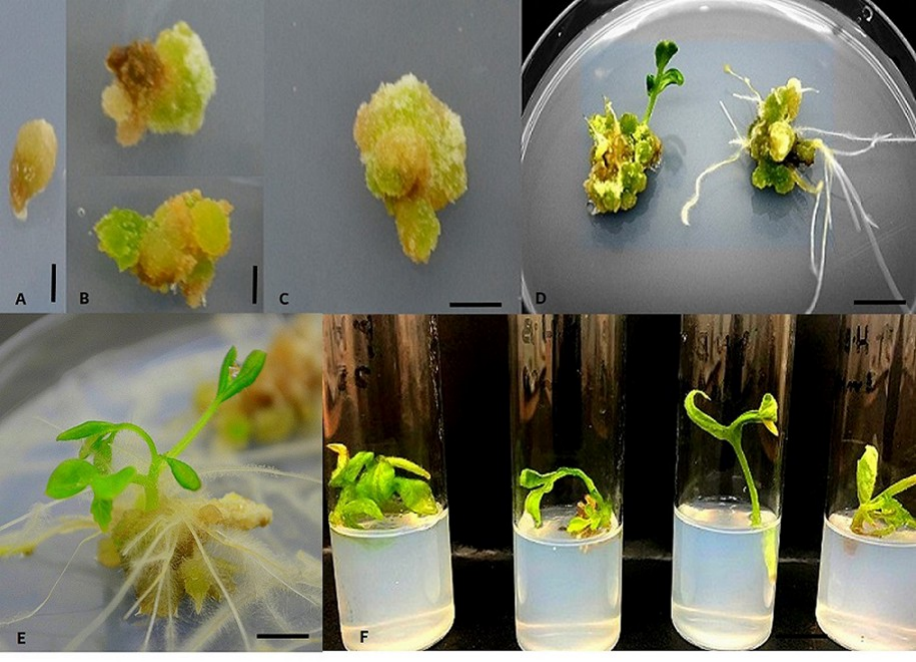
Induction of RTBs from rhizoids on MS media supplemented with TDZ/BAP (5 mg/L) at pH 4.0. (A) Individual rhizoids tuber excised from rhizoid cluster at pH 4.0. (B-C) Globular and heart shaped embryo development at pH 4.0. (D-F) *In vitro* germination of somatic embryos to multiple shoot and roots after 2 weeks of incubation at pH 5.8. Scale bar (A) 2 mm. Scale bars (B, C and E) 15 mm. Scale bar (D) 150 mm. Scale bar (F) 20 mm.

**Fig 4:**
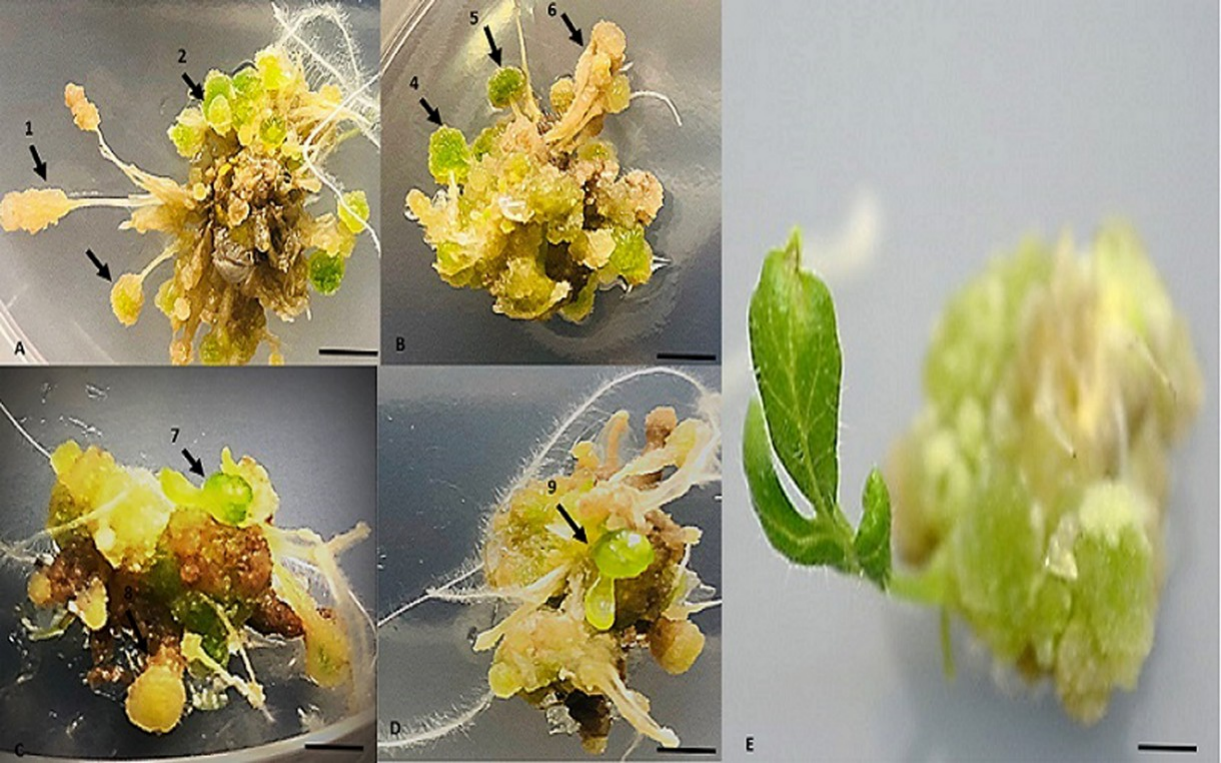
Stages of whole plantlet development from cluster of somatic embryos development via RTBs formation on MS media supplemented with TDZ at pH 4.0. (A-B) Induction of RTBs in light conditions, arrows showing development of club shaped structures. (C-D) Maturation of RTBs to pre-cotyledonary stage embryo at the tip of rhizoid cluster. (E) *In vivo* germination of somatic embryos to whole plantlet. Scale bars (A-E) 20 mm.

Rhizoids were allowed to mature at pH 4.0 on same medium and some of cluster rhizoids with many proembryos were shifted to a MS media supplemented with 5, 10, 15 or 20 mg/LTDZ and 5 mg BAP at for RTB induction. After 12 days of inoculation on rhizoid tuber induction medium (S2 Table) under light conditions (16/8 h, light/dark, white fluorescent lights 80 μmolm^-2^s^-1^), individual hair like rhizoids started formation of club shaped clusters termed as: rhizoid tubers (RTB) (Fig 3A). Contrary to photoperiod condition, the rhizoids left in dark lead to callus formation. It was found that RTBs were induced only on pH 4.0, while no such structures were observed at pH 5.8 (S1 Fig). The result analysis indicated that pH 4.0 satisfies formation of both rhizoids and RTBs. Addition of TDZ further enhanced the process of embryogenesis and many globular and torpedo- and heart shaped somatic embryos became visible after 3 days of incubation (Fig 4A–4D). RTBs were seen on both ends of explants in contact with medium while, central parts mainly showed progression through globular embryos, heart-torpedo and pre-cotyledonary embryo at the tip: as prospective shoot tip. Former were excised and allowed for *in vitro* shooting while, later formed well-developed shoot tip and vivo germination of embryo (Figs 3 and 4).

### Whole plantlet regeneration from RTBs

When different concentration of TDZ or BAP were supplemented in MS medium at pH 4.0 and 5.8, secondary somatic embryos at the surface of explants appeared. With increasing concentration of TDZ (20 mg/L) only somatic embryos were formed and number of pro-tubers and mature rhizoid tubers declines at pH 4.0. However, when explants were cultivated on medium with TDZ or BAP (5 mg/L) at pH 4.0, maximum number of RTBs was formed in first week, whereas, globular and heart torpedo shaped became visible (Fig 4A, 4B and 4C). When further incubation occurs under light conditions all primary embryos were converted to secondary embryos and shoot tip appeared at the top of explant (Fig 4D). These pre-cotyledonary embryos were distinctly different from primary somatic embryos. The cluster of RTBs formed adventitious shoots directly when cultured on TDZ or BAP (5 mg/L) at pH 5.8 (Fig 4E). Whereas, different stages of somatic embryos: globular and heart torpedo stages on the surface of explants were more prominent at pH 4.0. These embryos germinated like typical SE, an apical shoot developed first and sequentially leaves appeared later (Fig 4D and 4E). Induction of the shoots was exceptionally high on pH 5.8 in comparison to shoot morphogenesis, which was found to be slow at a lower pH 4.0 (Fig 3). This suggests that a lower pH with auxins (NAA) is required for rhizoid induction in the dark. Cytokinins (TDZ/BAP) addition in media with lower pH under light conditions induced novel structures–rhizoid tubers (RTBs) in *S*. *lycopersicum* cv. *Riogrande* (Tables 3 and 4) (Fig 4A and 4B). However, *in vitro* regeneration from RTBs was more favorable at pH 5.8. The low H^+^ concentration of growth media remained ubiquitous in tomato tissue culture in this study for induction of rhizoids, somatic embryos and RTBs formation. RTB embryoids were found to be novel structures consist of embryonic cells that spontaneously develop to form multiple plantlets in 2 weeks. Thus, a new regeneration system for fast and efficient propagation was optimized.

**Table 3:** Effect of TDZ/BAP concentration on rhizoid tubers (RTBs) induction at low pH in *S*. *lycopersicum* cv. *Riogrande* (no distinction of explant type).

TDZ mg/L	pH	Mean No. of RTBs /explant ±S.E	BAP mg/L	Mean No. of RTBs /explant ±S.E	Induction time
5	4.0	45.75±1.25^a^	5	44.5± 3.41^a^	12 Days
10	4.0	30.50±3.22^b^	—	—	12 Days
15	4.0	18.25±1.97^c^	—	—	12 Days
20	4.0	14.00±2.08^cd^	—	—	12 Days

Data represent the mean (no. of RTBs) ± standard error calculated from 120 explants in four replicates for each treatment.

Means followed by the same letter within column are not significantly different as determined by a pairwise comparison using Tukey’s (HSD) test at p<0.05.

TDZ: N-phenyl-N′-1, 2, 3-thiadiazol-5-ylurea.

**Table 4:** Effect of pH values on development of rhizoid tubers (RTBs) irrespective of explant type in *S*. *lycopersicum* cv. *Riogrande* supplemented with 5 mg/L of TDZ.

TDZ mg/L	pH value	Mean no. of RTBs /explant ±S.E
5	3.0	0.00±00^e^
5	4.0	46.35±0.05^a^
5	5.0	15.5±0.08^b^
5	6.0	7.94±0.02^c^
5	7.0	2.16±0.07^d^

Data represent the mean (no. of RTBs) ± standard error calculated from 120 explants in four replicates of each treatment.

Means followed by the same letter within column are not significantly different as determined by a pairwise comparison using Tukey’s (HSD) test at p<0.05.

TDZ: N-phenyl-N′-1, 2, 3-thiadiazol-5-ylurea.

#### Shoot organogenesis from calli

The dose of cytokinins alone as well as their combination with auxins has been found critical for shoot organogenesis in tomato. Adventitious shoot formation was reported to be highly dependent on type of explants as well as concentration of growth regulators [52]. Therefore, in this study we evaluated explant type, cultivar and treatment for regeneration of four tomato varieties. BAP alone at 3–5 mg/L induced more adventitious shoots and greater shooting percentage for all four varieties. Statistically significant differences were observed among shoot organogenesis between genotypes and explant types. The maximum numbers of shoots were induced on SIMT6 media with BAP (3 mg/L) and IAA (0.1 mg/L) with 4–5 shoots produced per explant of cv. *Riogrande* Fig 1G). BAP alone at 3–5 mg/L was found second most effective medium (SIMT3) in shoot regeneration. *Cv*. *Riogrande* showed 56.9% shoot induction frequency for cotyledon-derived explants at SIMT6. Significant differences were observed among all treatments at (p<0.05); all four cultivars depicted differential response to each treatment. The order of varietal shoot-induction frequency from cotyledons was *Rio>Roma>Hybrid>M82*. The effect of treatment ×genotype was found highly significant (Table 5). The time for morphogenesis of shoots ranged from 3.5 for SIMT6 and SIMT3 to 6 weeks for complete shoot organogenesis.

**Table 5:** Effects of culture media and explant type on shoot regeneration in four *S*. *lycopersicum* cultivars.

**Treatment/** **cv *Riogrande***	**BAP (mg/L)**	**NAA (mg/L)**	**IAA (mg/L)**	**KIN (mg/L)**	**Percentage of explants with Shoots± S.E**		**Mean no. of shoots± S.E** **No distinction of explant type**
					**Cotyledon**	**Hypocotyl**	
SIMT1	2	—	—	—	37.65±0.71^e^	35.01±0.81^c^	1.00±0.81 ^ab^
SIMT2	3	—	—	—	43.66±0.83^c^	40.23±0.76^b^	1.51±0.34^ab^
SIMT3	5	—	—	—	47.69±1.38^ab^	30.74±2.0^b^	3.48±0.21^a^
SIMT4	1		0.1	2	41.9348^d^	25.94±0.93^d^	1.05±0.61^ab^
SIMT5	3	0.1	—	—	48.25±0.83^ab^	45.67±0.36^a^	2.50 ± 1.0^a^
SIMT6	3	—	0.1	—	56.18±1.28^a^	43.68±1.9^a^	5.55±1.0^a^
**Treatment/ cv Roma**	**BAP (mg/L)**	**NAA (mg/L)**	**IAA (mg/L)**	**KIN (mg/L)**	**Percentage of explants with Shoots ± S.E**		**Mean no. of shoots± S.E** **No distinction of explant type**
					**Cotyledon**	**Hypocotyl**	
SIMT1	2	—	—	—	35.05±0.93^d^	33.05±2.09^c^	0.55±1.0^ab^
SIMT2	3	—	—	—	39.23±0.67^c^	30.98±0.72^d^	0.88±0.19^ab^
SIMT3	5	—	—	—	52.74±1.04^a^	44.74±2.7^ab^	3.00±0.16 ^a^
SIMT4	1		0.1	2	31.94±0.78^e^	34.94±0.73^c^	1.55±0.21^ab^
SIMT5	3	0.1	—	—	47.67±0.83^b^	46.90±0.836^a^	2.00±1.03 ^a^
SIMT6	3	—	0.1	—	47.68±1.49 ^b^	44.67±1.49^ab^	4.34±0.51 ^a^
**Treatment/ cv hybrid**	**BAP (mg/L)**	**NAA (mg/L)**	**IAA (mg/L)**	**KIN (mg/L)**	**Percentage of explants with Shoots ± S.E**		**Mean no. of shoots± S.E** **No distinction of explant type**
					**Cotyledon**	**Hypocotyl**	
SIMT1	2	—	—	—	29.99±1.03^d^	30.05±2.09^c^	0.65±0.11^a^
SIMT2	3	—	—	—	39.22±0.6^c^	33.98±0.72^b^	1.10±0.51^a^
SIMT3	5	—	—	—	40.20±1.08^a^	38.74±2.7 ^a^	2.6±0.89 ^a^
SIMT4	1		0.1	2	30.39±1.52^d^	30.94±0.73^c^	1.23±0.23^a^
SIMT5	3	0.1	—	—	28.61±1.40^de^	30.90±0.83^bc^	1.8±0.16 ^a^
SIMT6	3	—	0.1	—	34.61±1.80^b^	32.67±1.49^bc^	2.00±0.41 ^a^
**Treatment/ cv M82**	**BAP (mg/L)**	**NAA (mg/L)**	**IAA (mg/L)**	**KIN (mg/L)**	**Percentage of explants with Shoots ± S.E**		**Mean No. of shoots ± S.E** **No distinction of explant type**
					**Cotyledon**	**Hypocotyl**	
SIMT1	2	—	—	—	17.70±0.63^b^	13.05±1.09^c^	0.33±0.21^ab^
SIMT2	3	—	—	—	16.06±0.51^c^	13.28±1.72^c^	1.11±0.36 ^a^
SIMT3	5	—	—	—	21.39±2.10^a^	15.24±0.7^b^	1.5±0.18 ^a^
SIMT4	1		0.1	2	13.13±0.81^d^	15.24±1.73^b^	1.07±0.19 ^a^
SIMT5	3	0.1	—	—	16.53±0.21^c^	18.20±0.836^a^	0.55±0.3 ^ab^
SIMT6	3	—	0.1	—	13.67±0.50^d^	10.67±1.49^e^	0.72±0.16 ^a^

Data represent the mean (no. of explants with shoots /total no. of inoculated explants x100) ± standard error calculated from 200 explants

SIM: Shoot induction medium

Means followed by the same letter within column are not significantly different as determined by a pairwise comparison using Tukey’s (HSD) test at p<0.05.

The newly regenerated shoots were excised from callus interface and cultivated on root induction media (RIM) containing different hormones (Table 6). Rooting was observed 2 weeks post inoculation. RIM 5 with either NAA 0.1 mg/L or 0.5 mg/L and RIM 6 containing IBA 1 mg/L rendered a maximum number of roots. cv. *Riogrande* was most profound in rooting response with 12 roots/shoot on RIM 5. Irrespective of treatment cv. *Riogrande* and *Roma* showed 100 percent rooting frequency. However, differences in number and extent of rooting were seen. Well-rooted plantlets were hardened by shifting to soil substrate over a month before transferring them to natural environment. More than 90% of the plants survived with normal physiology with abilities to develop flowers and fruit.

**Table 6:** Effect of auxins on rooting of *in vitro* regenerating shoots of four *S*. *lycopersicum* cultivars after 8–10 weeks of incubation.

Treatment	IBA (mg/ L)	NAA (mg/L)	IAA (mg/L)	Mean no. of Roots± S.E cv Riogrande	Mean no. of Roots± S.E cv Roma	Mean no. of Roots± S.E cv Hybrid	Mean no. of Roots± S.E cv M82
RIMT1	0.1	—	—	6.16±0.7^c^	4.66±0.57^c^	3.33±0.57^c^	1.33±0.77 ^b^
RIMT2	0.2	—	—	4.66±2.08 ^c^	5.33±1.15^d^	2.33±0.57^d^	0.33±0.57^d^
RIMT3	0.5	—	—	8.0±0.50 ^ab^	4.66±1.52^c^	4.0±1.00 ^b^	1.66±1.5 ^b^
RIMT4	1	—	—	9.6±1.52 ^b^	6.33±1.52 ^b^	2.0±1.00 ^e^	4.0±1.00 ^a^
RIMT5	—	0.5/0.1	—	12.6±2.08^a^	10.33±1.52^a^	7.33±0.57^a^	1.0±1.00 ^c^
RIMT6	—	—	0.1	7.0±1.00^ab^	5.66±2.08^c^	4.66±1.52 ^b^	1.33±1.52^b^
RIMT7			0.2	6.66±1.52^c^	4.66±2.08 ^c^	2.33±0.57^d^	1.33±0.57 ^b^

Data represent the mean (no. of roots developed from each shoot) ±standard error of three replications calculated from 200 explants for each treatment.

RIM: Root induction medium

Means followed by the same letter within column are not significantly different as determined by a pairwise comparison using Tukey’s (HSD) test at p<0.05.

### Histological analysis of RTBs revealed proembryos and multiple embryoids

**Fig 5:**
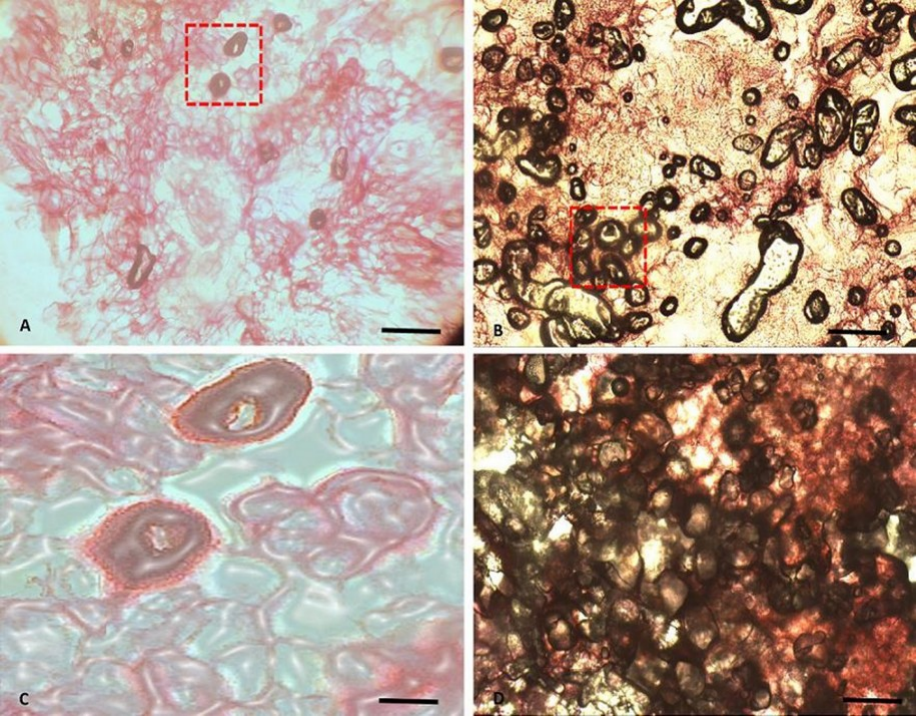
Light microscopic sections of rhizoid tubers (RTBs) stained with safranin stain on tuber induction medium supplemented with 5 mg/L TDZ at pH 4.0 from cotyledon explants of *S*. *lycopersicum* cv. *Riogrande*. (A) Section of regenerating rhizoid tubers after 25 days of incubation showing globular and nodular embryos arising from aggregate of callus tissues, 40 X. (B) Enlarged view of T-section showing embryonic cells. (C) Transverse section of mature RTB, 20X automatic scanning system [ASS]. (D) Enlarged view of section under box showing multiple embryoids. Scale bars (A, C) 200 μm. scale bars (B, D) 50 μm.

**Fig 6:**
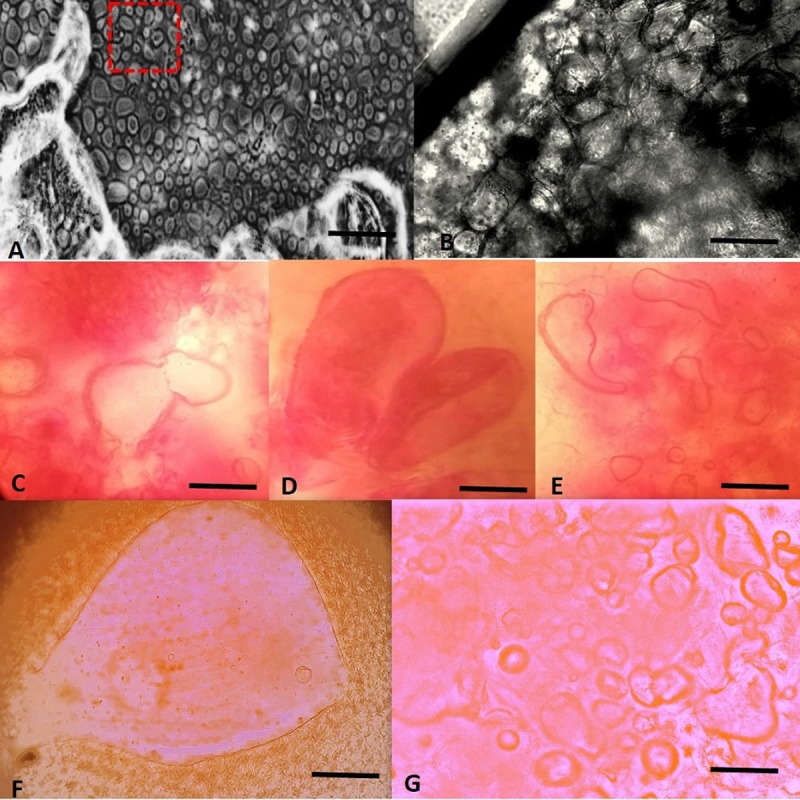
Histology of somatic embryos developed via direct somatic embryogenesis from cotyledons explants of *S*. *lycopersicum* cv. *Riogrande*. (A) Section of explants with various stages of somatic embryos showing abundance of globular stage embryos, after one week on tuber induction medium. (B) Enlarged view of transverse section under red box. (C) Globular shaped embryos [nucleus not stained] (D) Heart shaped embryos. (E) Torpedo-shaped cells. (F) Longitudinal section of single RTB club shaped embryo attached to explant. (G) Safranin stained section showing different stages of SE scale bars (A, G) 200 μm. scale bars (C, D, E) 200 μm. Scale bar (B, F) 50 μm.

Microscopic observation of the transverse section of mature RTBs and calli containing embryos stained with safranin showed an internal arrangement of embryonic cells and non-embryonic cells. After 12 days RTBs cultivation on TIM, rhizoid pro-embryos accepted the dye and turned visibly dark pink, while non-embryonic tissues remained unstained (Fig 5A–5D). The embryonic tissues were distinctly stained pinkish red with globular, nodular, bi-lobed heart shaped and cotyledon shaped stages (Fig 6C, 6D and 6E). Each tuber exhibited multiple embryoids at different stages of development, hence progressing like typical somatic embryogenesis to a whole plantlet (Fig 6A and 6B).

## Discussion

Conventional breeding management augmented with sustainable *in vitro* cell cultures can be exploited for the production of genetically engineered plants. The development of this system will be of great value in germplasm conservation, somatic embryogenesis, and the multiplication of *in vitro* grown seedlings.

This research focuses on comparisons of *in vitro* morphogenesis in four tomato cultivars via organogenesis through callus induction and direct somatic embryogenesis (SE). For this purpose, we optimized the germination and sterilization protocols of all the cultivars of tomato to increase their efficiency for *in vitro* regeneration. The use of NaOCl has been proved to be effective in maintaining uniform tomato cell culture [52,53]. *In vitro* germination of tomato seeds observed to be highest for cv. *Riogrande* and *Roma* after using 6% NaOCl with and without tween 20 as surfactant. The results are in line with [54,55] who have used similar treatment for *in vitro* germination of tomato seeds.

### Conventional morphogenesis of *Solanum lycopersicum L*

*In vitro* morphogenesis response of the different tomato cultivars relies on genotype × environmental factors, thus making tomato transformation complex. Different concentrations of PGRs were used alone and in combination to evaluate *in vitro* regeneration response. Explants (one week) were used for callus induction on 16 different media compositions, which showed that higher cytokinins with low auxins tend to induce callus formation. Highest callus induction was observed from the MS basal medium containing 2 mg/L IAA, 2 mg/L NAA, 2 mg/LBAP, 4 mg/L KIN (CIMT^12^) in both young cotyledons and hypocotyls of *Riogrande* and *Roma* where callus induction index was >80%. While *M82* and hybrid line *17905* responded to a completely different set of PGRs owing to genotypic differences. Such observations are in line with previous reports, where various combinations of PGRs were found cultivar-dependent and accounted as a trivial cause of slow *in vitro* regeneration.

Our results are consistent with [16,56–58] who reported that the regeneration capacity of juvenile explants is better as compare to adult explants. Our findings also suggest the superiority of cotyledons over hypocotyls in terms of callus formation and regeneration (Fig 1). Embryogenic calli derived from cotyledon explants depicted a high-shoot regeneration potential in *Riogrande* and *Roma* while the other two cultivars had pale white compact calli indicative of slow organogenesis. Hypocotyl derived calli have many embryoids in *Riogrande*, however, callus induction was slower and calli were less totipotent as compared to cotyledons.

Various shoot induction treatments containing BAP at high concentration with or without low dose of auxins were evaluated for *in vitro* shoot formation and number of shoot primordia per calli. Regenerating calli led to multiple shoot formations in a 3-week time period on SIMT6 containing 3 mg/L BAP, 0.1 mg/L IAA. On an average, 4–5 shoots were induced on SIM in case of *Riogrande* with 56% shooting frequency followed by *Roma*, *hybrid 17905* and *M82*. BAP alone at 5 mg/L was found second most effective treatment in terms of shoot organogenesis. These findings corroborates with reports of using high cytokinins particularly BAP for short-time shoot multiplication in different tomato cultivars [59,60]. Previously, it has been reported that regeneration response is highly genotype dependent and combination of PGRs working for one cultivar may not be suitable for other cultivars [13,24]. Our results have shown superiority of cv. *Riogrande* and *Roma* over *hybrid 17905* and *M82* with respect to *in vitro* regeneration.

Individual regenerated shoots (3–4 cm) were excised and subsequently cultured on rooting medium containing different levels of IAA, NAA, and IBA. There after highest number of roots (~12) were observed from shoots of cv. *Riogrande* with 0.1 or 0.5 mg/L of NAA in basal medium followed by cv. *Roma* (~10). IBA at 1 mg/L was found second most effective rooting hormone. These findings could be explained by positive effect of exogenous auxins on root development [61]. Our results are in line with [62] who used IBA and IAA for more than 10 roots/shoot in tomato.

**Fig 7:**
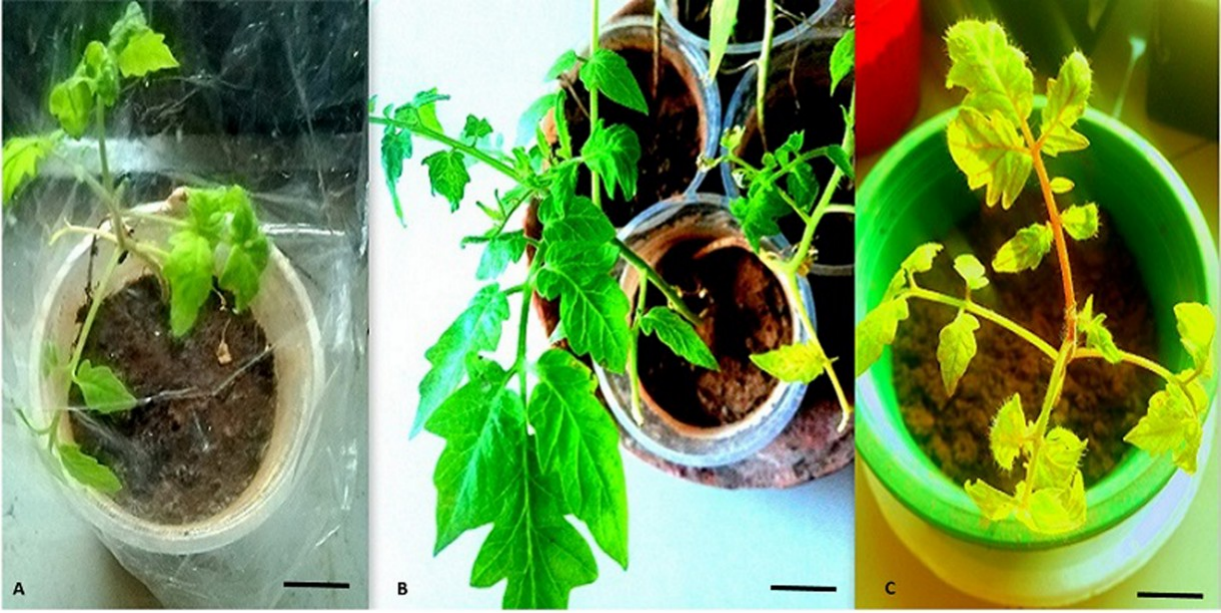
Plant acclimatization and transferring to soil substrate. (A) 2 months old, plants transferred to soil with plastic cover in growth room. (B-C) Plants transferred to natural environment. Scale bar (A, B, C) 2 cm.

Our present experiments indicated the superiority of cv. *Riogrande* and *Roma* over *hybrid 17905* and *M82* with respect to *in vitro* regeneration. Whole organogenesis process completed in less than 2.5 months for cv. *Riogrande* with 3 weeks of callogenesis, 3 weeks for shoot multiplication, and 12 days of rooting. This high frequency standardized procedure yields acclimatized plantlets in 2–3 months (Fig 7).

### Lower pH mediated *in vitro* regeneration though somatic embryogenesis

To further, shorten the time of *in vitro* plantlet regeneration, organogenesis via SE in cv. *Riogrande* was developed. The effect of growth medium’s pH on SE is reported here as a novel aspect of tomato regeneration which influences the overall progress of clonal propagation and cell cultures. SE in tomato was achieved in two steps by induction of rhizoid and rhizoid tubers. In the first step, rhizoids were induced form a week-old cotyledons and hypocotyls by using two auxin analogues at various concentration (NAA & 2, 4-D) in dark conditions at pH 4.0. NAA concentration at 0.5 or 2 mg/L promoted substantial rhizoid induction while 2,4-D failed to induce these structures. The role of auxin to promote induction phase in SE is well documented. Use of different auxins like IAA, IBA, NAA and a highly active analogue 2,4-D are considered to be the most important hormones that help in regulation of auxin gradient during SE. In particular, the embryogenic cultures are induced by the little quantity of auxins in the culture medium while increase in concentration of auxins favors callus formation. Embryogenesis primarily rely on manipulation of PGRs irrespective of plant species, however, competence of particular type of explant towards exogenous hormones is largely dependent on genotype and is more like hit and trail method. Unlike many previous reports of using 2,4-D for initiation of S.E, the results reported here are contradictory [63]. No embryogenesis was observed when explants were exposed to varying concentration of 2,4-D at pH 4 (Table 2). In a study done on sweet potato, treatment of calli with 5 μM 2,4-D and polar auxins transport inhibitor 2,3,5-triiodobenzoic acid restricted the development of embryo promoting only callus morphogenesis. Which showed that exogenous phytohormones particularly auxins effect endogenous polar IAA transport to facilitate or inhabit embryo development [64].

Due to presence of particular type of auxins in the culture medium, NAA in our study, pro-embryogenic masses already present in the culture are primed to undergo primary SE and initiation of cell polarity occurs. Thus, initiation phase began by one of two auxins. Once the auxins were removed from the culture, embryogenesis proceeded rapidly. Such observation have been reported in many plant species [65]. Thus, individual rhizoids, upon transfer to a medium containing 5 mg/L TDZ or BAP in light conditions, produced secondary embryogenesis and novel structures–rhizoid tubers (RTBs) with many somatic embryoids (Fig 4). Different novel structures like RTBs, frog- egg-, and bulbil-like bodies have been reported through SE recently [66–68] and our findings are in agreement with the previous reports [69]. TDZ at high concentration not only induced RTBs but also germination of embryos to cotyledonary nodes on top of explant. When TDZ was used for *in vitro* plantlet regeneration of excised RTBs, they spontaneously germinated to shoot primordia without any intermediated stages. TDZ have been known as potent inducer of de-differentiation in diverse plant species where it mimics cytokinins like activity and change the level of endogenous auxins. The morphogenetic responses are modulated by high concentration of TDZ that may constitute inductive signal for embryogenic expression [70,71]. The results are in line with [72,73] where combination of TDZ and BAP at high concentration resulted in somatic embryos while sequential incubation on BAP followed by TDZ, shoot organogenesis occurred. By simply changing or alternating two cytokinins, shift from somatic embryos to shoot formation could be achieved for RTBs. The embryogenic response of the different species also appear to depict varying level of sensitivity towards PGRs [33]. The specific requirements to determine the fate of de-differentiation operate not only at specie level but also on cultivar level [74]. Hence forth, among same species embryogenetic competency may vary against different PGRs and cell status and endogenous hormone level [63].

TDZ and BAP at 5 mg/L in light not only favored formation of RTBs but also *in vivo* shoot organogenesis occurred when RTBs from TDZ were transferred to medium with BAP (Table 3).

Further these RTBs originated as secondary somatic embryos from primary ones and they consist of multiple embryonic cells hence, multiple embryo formation within RTBs occurs. Eventually these embryos germinate spontaneously and are capable of high frequency *in vitro* shoot formation (Fig 3). These results demonstrate the uniqueness of novel structures—RTBs for complete organogenesis in less time and labor over routinely used explants sources.

Illumination also played a significant role in the SE of tomato. Dark conditions favored induction of rhizoids while light enhanced RTBs formation. Studies have shown the positive effect of light treatment on SE of different plant species [75,76]. However, continuous photoperiod has been proven to affect the cell culture viability by production of inhibitory compounds that led to browning of explants, hence, reduced light exposure favors S.E in carrot cultures [77]. Unlike many reports of continuous dark regime, it is noteworthy that induction of SE was initiated in dark conditions while maturation of RTBs was more prominent in light photoperiod [78]. We here report sequential dark and light impulses for quick embryogenesis response at pH 4.0.

During SE, MS medium supplemented with a high level of auxins (2 mg/L NAA) and pH 4.0, not only limited yeast contamination but also shortened the time of somatic embryogenesis and regeneration. A significant number of rhizoids were produced on MS medium containing NAA at pH 4 while higher pH values resulted in callogenesis rather than rhizoids induction. To our knowledge, there is no report of *in vitro* regeneration in tomato at pH 4.0. However, low pH has been used to grow cell cultures of other plants. An increased number of regenerated shoots at lower pH (4.5) in *Bacopa monnieri* has been reported [79]. pH range (3–7) was used to evaluate the regeneration capacity in pine buds showing that initially low pH of media is more suitable for the *in vitro* morphogenesis [80]. Consistent with this idea low medium pH also impose tissue culture impulses and may be perceived as a stress signal, hence forcing the reprogramming of cell dedifferentiation to embryogenesis in cv. *Riogrande*. Such notion is supported by [81] where increase in xylem pH has been associated with elevated ABA concentration in the apoplastic region adjacent to guard cell of leaf epidermis. The medium pH tend to affect nutrient availability as well as enzymatic and hormonal activities in tomato thus increasing shoot biomass in cv. *Redcoat* [82]. However, contrary to previous observation pH 4.0 with specific auxins at low concentration favors rapid somatic embryogenesis by formation of rhizoids and RTBs, while higher concentration of auxins at pH 4.0 led to callogenesis. Here we report the low pH as novel aspect of embryogenesis competency in cv. *Riogrande* that wasn’t explored previously.

Embryogenesis is demonstrated as a process consisting of callusing and redetermination of cells [83]. Thus, implying that secondary embryogenesis may or may not proceed with callus formation. The RTBs were seen as somatic embryos consisting of many embryoids as well as embryonic cells. Histological studies of RTBs demonstrated their composition of embryogenic cells with multiple embryonic stages (Figs 6 and S5). These embryos sprout from epidermis of rhizoids and then develop into whole plantlets through various growth stages. Most of embryonic cells were found thick walled, with distinctly stained cytoplasm.

The highlighted findings of experiments suggest that the selection of right auxins at low pH (i.e. 4.0) along with alternate incubation in dark/light photoperiod conditions are favorable conditions for the induction of SE. The pH values < 4.0 were unable to support SE while > 4.0 led to callus induction. These rhizoids were first induced from the cotyledon explants in the dark, which later developed into rhizoid tubers in light conditions via SE. The presence of embryonic cells inside individual RTBs indicates their ability to form multiple embryos. As the primary embryos were converted to secondary embryos, the pre-cotyledonary node appeared on same induction medium leading to *in vivo* shoot organogenesis (Fig 4). While the excised rhizoid tubers also matured to *in vitro* shoot and root formation (Fig 3). The whole procedure of embryogenesis was completed in initiation phase (6 days), maturation and secondary embryogenesis (6 days), shoot and root morphogenesis (15 days), shoot proliferation and acclimatization of plantlets (12 days). Thus, the process following germination of one-week old seedling, initiation of embryogenesis and subsequent germination of cotyledonary embryos to whole plantlet was completed in 45 days. Large number of somatic embryos were produced from single explant though this protocol in a short time, which is critical factor for successful *in vitro* culture for most recalcitrant genotypes. Such system has been previously reported in tobacco leaf explants (model system) by inducing direct SE pathway in 6 days [84]. While in tomato the average time for somatic embryogenesis response was reported to be 4–6 weeks [36,38]. The merits of such robust system involving whole plantlet generation via SE as potential model to study molecular and regulatory events in plant development and endogenous phytohormones regulation cannot be overlooked in tomato as discussed by [74]. The existing reports of stress inductive regulatory role of novel phytohormones provide vistas for transcriptional level regulation of S.E [85].

## Conclusion

Considering the commercial and economic importance of Rio tomatoes, the regeneration system via SE involving newly identified structures (RTBs) in tomato will enhance the introduction of economically important traits in transgenic plants as it can yield multiple plantlets of tomato from single explant in 45 days. In many plant species induction of SE and recovery of viable plantlets is not frequently achievable. Nonetheless, the connotation SE has gained importance in clonal propagation as a useful tool to be used instead of conventional micro-propagation. While secondary embryos serve as excellent choice of explant for gene transfer technology. In addition, the RTBs and SE regeneration system could be used to overcome recalcitrance phenomenon in tissue culture and transformation of various other varieties of tomato. The results obtained are relevant for speed up of breeding program by production of large scale true to type plants of tomato lines with desired characteristics. Such system can be applied to predict the embryogenic competence of particular lines and their subsequent use for transformation and large-scale propagation. The RTBs will be available as a juvenile source of explant with high capacity to yield plantlets. Further, dedicated studies can confirm ubiquity of RTBs as secondary embryos and *in vitro* regeneration in other plant species as well.

## Supporting information

S1 Table Effect of clorox (NaOCl) concentration on sterilization of seeds of *Solanum lycopersicum* L. cvs. *Riogrande*, *Roma*, *M82* and *hybrid* (*17905*) on full and ½MS medium without sucrose. Tomato seeds of all varieties sterilized with >10% sodium hypochlorite exhibited complete loss of germination activity irrespective of media composition. While 6% sodium hypochlorite and 8% house hold bleach, treatment followed by 2 days incubation in dark was found most optimal for seed germination on half strength and full-strength medium. Lower level of sodium hypochlorite (1–2.5%) was found to induce early germination however, 50% of the cultured seeds were infected with fungal contamination. Among different treatments applied for seed disinfection, 6% NaOCl for 15 min with gentle agitation and 8% house hold bleach for 10 min was found most significant to achieve aseptic germination on full strength and half-strength (½) Murashige and Skoog medium after 6 days of incubation. The use of tween 20 in combination with NaOCl was suboptimal. The four varieties tested in this study showed slightly more but not significantly different germination activity on half strength MS media as compared to full strength. Seeds of cv. *Riogrande* and *Roma* showed 90–80% germination index on both media (P<0.05) however *Hybrid-17905* and *cv*. *M82* were significantly slower in germination response.
(DOCX)

S2 Table Optimized media formulations used for in vitro morphogenesis and transformation of *S*. *lycopersicum* cultivars. TDZ: N-phenyl-N′-1, 2, 3-thiadiazol-5-ylurea.
(DOCX)

S1 Fig Callus morphology of *Solanum lycopersicum* L. cvs. *Riogrande*, *Roma*, *M82* and *hybrid (17905)* irrespective of explant type. (A) Greenish soft calli induced from cotyledons of Rio. (B) Off white with green portions calli induced from cotyledons of Roma. (C) Off white calli with green spots induced from cotyledons of hybrid 17905. (D) Pale white calli induced from cotyledons of M82. Scale bars (A, B, C, D) 150 mm.
(PDF)

S2 Fig Effect of pH values on rhizoid formation cultured on MS media supplemented with 2mg/L NAA under dark conditions after one week. (A-C) Explants cultured on pH 4.0 + 2mg/L NAA. (C) Enlarged view under box showing hair like rhizoid extensions from explant edges. Explants cultured on pH 4.0 + 2mg/L NAA. (D) Explants cultured on pH 3.0 + 2mg/L NAA. (E) Explants cultured pH 5.0 + 2mg/L NAA in dark conditions. (F) Explants cultured pH 5.0 + 2mg/L NAA. (G) Explants cultured pH 5.0 + 2mg/L NAA in dark conditions. Scale bars (A, D, E, F, and G) 150 mm. Scale bar (B) 200 mm. Scale bar (C) 20 mm.
(PDF)

S3 Fig Rhizoids formation on pH 4.0 vs pH 5.8 under light and dark conditions after one week of incubation on rhizoid induction medium. (A) Explants cultured on pH 4.0 + 2mg/L NAA in light conditions. (B) Explants cultured on pH 4.0 + 2mg/L NAA in dark conditions. (C) Explants cultured on pH 5.8 + 2mg/L NAA in light conditions. (D) Explants cultured pH 5.8 + 2mg/L NAA in dark conditions. Scale bars (A-D) 150 mm.
(PDF)

S4 Fig Individual rhizoids excised and incubated on TDZ under light for *in vitro* shoot formation. Scale bar, 100mm.
(PDF)

S5 Fig **At an earlier stage, the cluster of rhizoid hair structure (A) was fixed and stained. (B) The cross-sectional image shows globular and bipolar embryos originating from sub-epidermal cells.** Scale bar (A) 20mm. Scale bar (B) 50μm.
(PDF)
