# Supplementary material for: Efficient and reproducible somatic embryogenesis and micropropagation in tomato via novel structures - Rhizoid Tubers
Source: PLoS One. 2019 May 22;14(5):e0215929. doi: 10.1371/journal.pone.0215929 (PMC6530835; doi:10.1371/journal.pone.0215929)
Supplement: S1 Table — Tomato seeds of all varieties sterilized with >10% sodium hypochlorite exhibited complete loss of germination activity irrespective of media composition. While 6% sodium hypochlorite and 8% house hold bleach, treatment followed by 2 days incubation in dark was found most optimal for seed germination on half strength and full-strength medium. Lower level of sodium hypochlorite (1–2.5%) was found to induce early germination however, 50% of the cultured seeds were infected with fungal contamination. Among different treatments applied for seed disinfection, 6% NaOCl for 15 min with gentle agitation and 8% house hold bleach for 10 min was found most significant to achieve aseptic germination on full strength and half-strength (½) Murashige and Skoog medium after 6 days of incubation. The use of tween 20 in combination with NaOCl was suboptimal. The four varieties tested in this study showed slightly more but not significantly different germination activity on half strength MS media as compared to full strength. Seeds of cv. Riogrande and Roma showed 90–80% germination index on both media (P<0.05) however Hybrid-17905 and cv. M82 were significantly slower in germination response. (DOCX) [file pone.0215929.s001.docx]

**Table S1_._ Effect of Clorox (NaOCl) concentration on sterilization of seeds of *Solanum lycopersicum L*. cvs. *Riogrande*, *Roma*, *M82* and *hybrid* (*17905*) on full and ½MS medium without sucrose.**

| NaOCl (% v/v) | House Hold bleach (% v/v) | Tween 20 | Treatment duration (min) | %Germination : %Contamination | | | | Remarks |
| --- | --- | --- | --- | --- | --- | --- | --- | --- |
|  |  |  |  | **Rio** | **Roma** | **Hybrid** | **M82** |  |
| 1 | - | - | 15 | 60: 90 | 63:80 | 69:89 | 66:78 | Contamination |
| 2 | - | - | 15 | 65:50 | 67:60 | 56:61 | 45:62.5 | >50 contamination |
| 3 | - | - | 15 | 87:20 | 85:22 | 60:25 | 35:25 | >50 contamination |
| 5 | - | - | 15 | 92:8 | 89:13 | 72:15 | 30:20 | Optimal germination |
| 6 | - | - | 15 | 95:5 | 93:7 | 82:12 | 30:5 | maximum germination |
| 6 | - | 2 drops | 15 | 85:20 | 76:20 | 66:25 | 25:2 | Sub optimal germination |
| 10 | - | - | 10 | 20:0 | 15:0 | 18:0 | 10:0 | <20% germination |
| 15 | - | - | 10 | 2:0 | 3:0 | - | - | No germination |
| 20 | - | - | 10 | 0 | 0 | - | - | No germination |
| - | 8 | - | 15 | 72:20 | 74:23 | 60:15 | 10:25 | Delayed germination |

Tomato seeds of all varieties sterilized with >10% sodium hypochlorite exhibited complete loss of germination activity irrespective of media composition. While 6% sodium hypochlorite and 8% house hold bleach, treatment followed by 2 days incubation in dark was found most optimal for seed germination on half strength and full-strength medium. Lower level of sodium hypochlorite (1-2.5%) was found to induce early germination however 50% of the cultured seeds were infected with fungal contamination. Among different treatments applied for seed disinfection, 6% NaOCl for 15 min with gentle agitation and 8% house hold bleach for 10 min was found most significant to achieve aseptic germination on full strength and half-strength (½) Murashige and Skoog medium after 6 days of incubation. The use of tween 20 in combination with NaOCl was suboptimal. The four varieties tested in this study showed slightly more but not significantly different germination activity on half strength MS media as compared to full strength. Seeds of cv. *Riogrande* and *Roma* showed 90-80% germination index on both media (P<0.05) however *Hybrid-17905* and *cv. M82* were significantly slower in germination response.
