# Supplementary material for: Efficient and reproducible somatic embryogenesis and micropropagation in tomato via novel structures - Rhizoid Tubers
Source: PLoS One. 2019 May 22;14(5):e0215929. doi: 10.1371/journal.pone.0215929 (PMC6530835; doi:10.1371/journal.pone.0215929)
Supplement: S2 Table — TDZ: N-phenyl-N′-1, 2, 3-thiadiazol-5-ylurea. (DOCX) [file pone.0215929.s002.docx]

**Table S2. Optimized media formulations used for in vitro morphogenesis and transformation of *S. lycopersicum* cultivars.**

| **Optimized medium used for regeneration and transformation of tomato** | |
| --- | --- |
| **Culture medium** | **Additional Components** |
| Germination medium (GM) | 3.17g/L MS salts, 8g/L plant Agar and pH 5.8 |
| Callus induction medium (CIM) | 4.15 g/ L, 20g/L sucrose, 8g/L plant Agar ,2mg/L NAA, 2mg/L IAA, 2mg/L BAP, 4mg/L KIN pH 5.8 or  4.15 g/ L, 20g/L sucrose, 4g/L Phytagel, 2mg/L NAA. 2mg/L IAA, 2mg/L BAP, 4mg/LZEA , and pH 5.8 (cv. M82) |
| Shoot induction Medium (SIM) | 4.15 g/ L MS salts, 20g/L sucrose, 8g/L plant Agar ,3mg/L BAP and 0.1mg/L IAA pH 5.8 |
| Root induction Medium (RIM) | 4.15 g/ L MS salts, 20g/L sucrose, 8g/L plant Agar and0.5mg/L NAA or 1mg/L IBA pH 5.8 |
| Rhizoids induction medium (RhIM) | 4.15 g/ L MS salts, 20g/L sucrose, 8g/L plant Agar, 0.5 or 2 mg/L NAA and pH 4.0 |
| Tubers induction Medium (TIM) | 4.15 g/ L MS salts, 20g/L sucrose, 8g/L plant Agar, 5mg/L BAP or 5mg/L TDZ and pH 4.0 |
| Pre-Culture Medium (PCM) | 4.15 g/ L MS salts, 20g/L sucrose, 8g/L plant Agar , 1mg/L NAA,1mg/L BA P pH 5.8 |
| Co-CultivationMedium (CCM) | 4.15 g/ L MS salts, 20g/L sucrose, 8g/L plant Agar,2mg/L NAA, 2mg/L IAA, 2mg/L BAP, 4mg/L KIN and 200 μM *Acetosyringone*, pH 5.8 |
| Selection Medium (SM) | 4.15 g/ L MS salts, 20g/L sucrose, 2mg/L IAA, 2mg/L BAP, 4mg/L KIN, 300mg/L Cefotaxime/ 300mg/L Augmentin, and 600 mg/L Ticarcillin pH 5.8 |
| Infiltration Medium (IFM) | 4.15 g/ L MS salts, 20g/L sucrose, 2mg/L NAA and 100 μM *Acetosyringone* |
| Inoculation Medium (IM) | 4.15 g/ L MS salts, 20g/ L sucrose and 200 μM *Acetosyringone* |
