# Supplementary material for: Efficient and reproducible somatic embryogenesis and micropropagation in tomato via novel structures - Rhizoid Tubers
Source: PLoS One. 2019 May 22;14(5):e0215929. doi: 10.1371/journal.pone.0215929 (PMC6530835; doi:10.1371/journal.pone.0215929)
Supplement: S1 Fig — (A) Greenish soft calli induced from cotyledons of Rio. (B) Off white with green portions calli induced from cotyledons of Roma. (C) Off white calli with green spots induced from cotyledons of hybrid 17905. (D) Pale white calli induced from cotyledons of M82. Scale bars (A, B, C, D) 150 mm. (PDF) [file pone.0215929.s003.pdf]

Fig S1. Callus morphology of *Solanum lycopersicum L.* cvs. *Riogrande*, *Roma*, *M82* and *hybrid (17905)* irrespective of explant type.

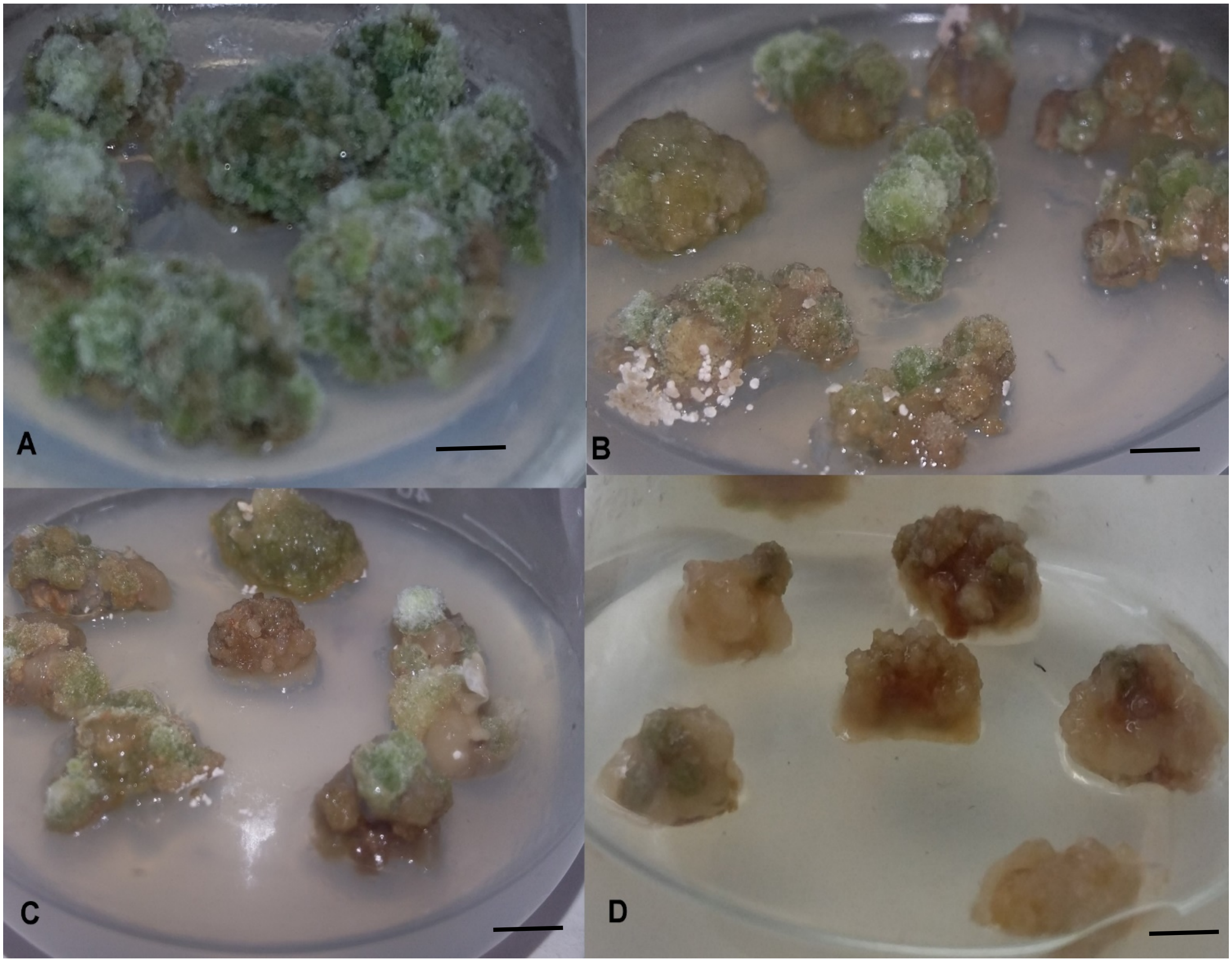

(A) Greenish soft calli induced from cotyledons of Rio. (B) Off white with green portions calli induced from cotyledons of Roma. (C) Off white calli with green spots induced from cotyledons of hybrid 17905. (D) Pale white calli induced from cotyledons of M82. Scale bars (A, B, C, D) 150 mm.
