# Supplementary material for: Efficient and reproducible somatic embryogenesis and micropropagation in tomato via novel structures - Rhizoid Tubers
Source: PLoS One. 2019 May 22;14(5):e0215929. doi: 10.1371/journal.pone.0215929 (PMC6530835; doi:10.1371/journal.pone.0215929)
Supplement: S2 Fig — (A-C) Explants cultured on pH 4.0 + 2mg/L NAA. (C) Enlarged view under box showing hair like rhizoid extensions from explant edges. Explants cultured on pH 4.0 + 2mg/L NAA. (D) Explants cultured on pH 3.0 + 2mg/L NAA. (E) Explants cultured pH 5.0 + 2mg/L NAA in dark conditions. (F) Explants cultured pH 5.0 + 2mg/L NAA. (G) Explants cultured pH 5.0 + 2mg/L NAA in dark conditions. Scale bars (A, D, E, F, and G) 150 mm. Scale bar (B) 200 mm. Scale bar (C) 20 mm. (PDF) [file pone.0215929.s004.pdf]

Fig S2. Effect of pH values on rhizoid formation cultured on MS media supplemented with 2mg/L NAA under dark conditions after one week.

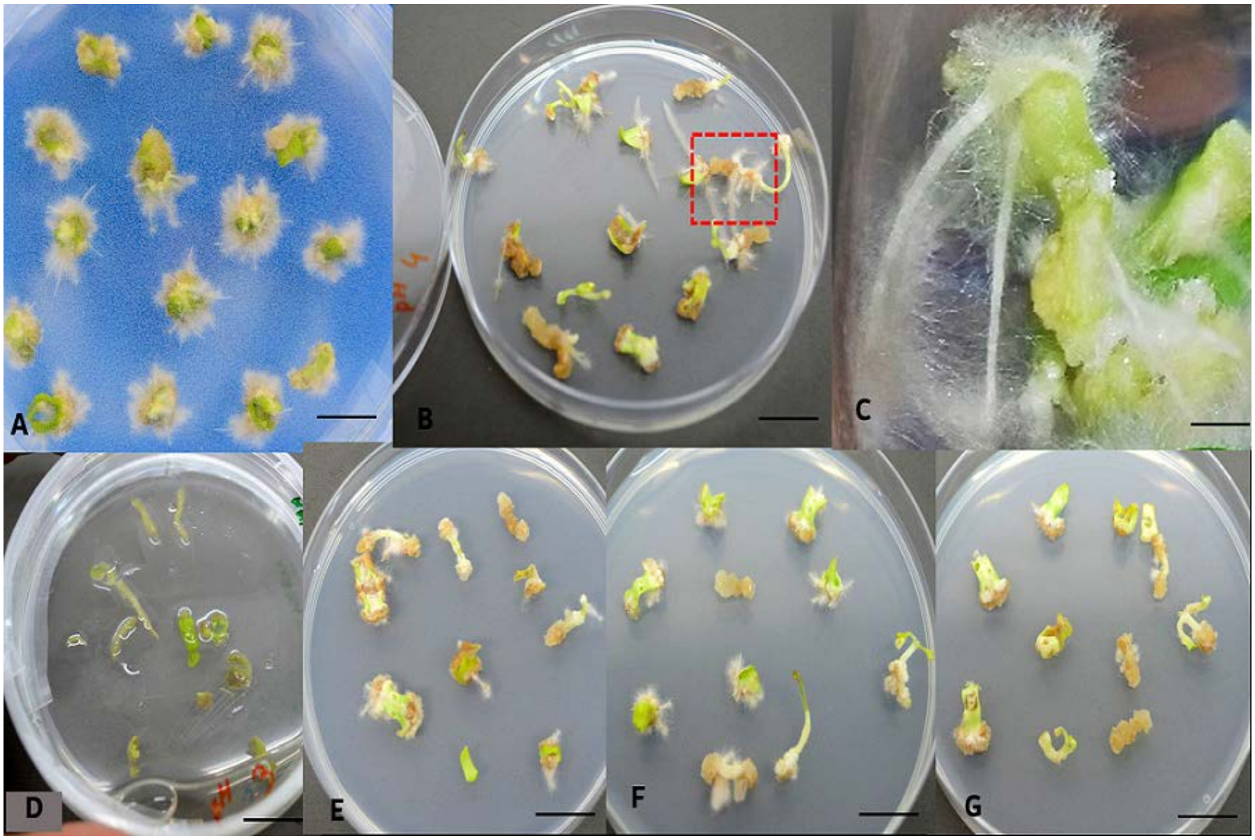

(A-C) Explants cultured on pH 4.0 + 2mg/L NAA. (C) Enlarged view under box showing hair like rhizoid extensions from explant edges. Explants cultured on pH 4.0 + 2mg/L NAA. (D) Explants cultured on pH 3.0 + 2mg/L NAA. (E) Explants cultured pH 5.0 + 2mg/L NAA in dark conditions. (F) Explants cultured pH 5.0 + 2mg/L NAA. (G) Explants cultured pH 5.0 + 2mg/L NAA in dark conditions. Scale bars (A, D, E, F, and G) 150 mm. Scale bar (B) 200 mm. Scale bar (C) 20 mm.
