# Supplementary material for: Efficient and reproducible somatic embryogenesis and micropropagation in tomato via novel structures - Rhizoid Tubers
Source: PLoS One. 2019 May 22;14(5):e0215929. doi: 10.1371/journal.pone.0215929 (PMC6530835; doi:10.1371/journal.pone.0215929)
Supplement: S3 Fig — (A) Explants cultured on pH 4.0 + 2mg/L NAA in light conditions. (B) Explants cultured on pH 4.0 + 2mg/L NAA in dark conditions. (C) Explants cultured on pH 5.8 + 2mg/L NAA in light conditions. (D) Explants cultured pH 5.8 + 2mg/L NAA in dark conditions. Scale bars (A-D) 150 mm. (PDF) [file pone.0215929.s005.pdf]

Fig S3. Rhizoids formation on pH 4.0 vs pH 5.8 under light and dark conditions after one week of incubation on rhizoid induction medium.

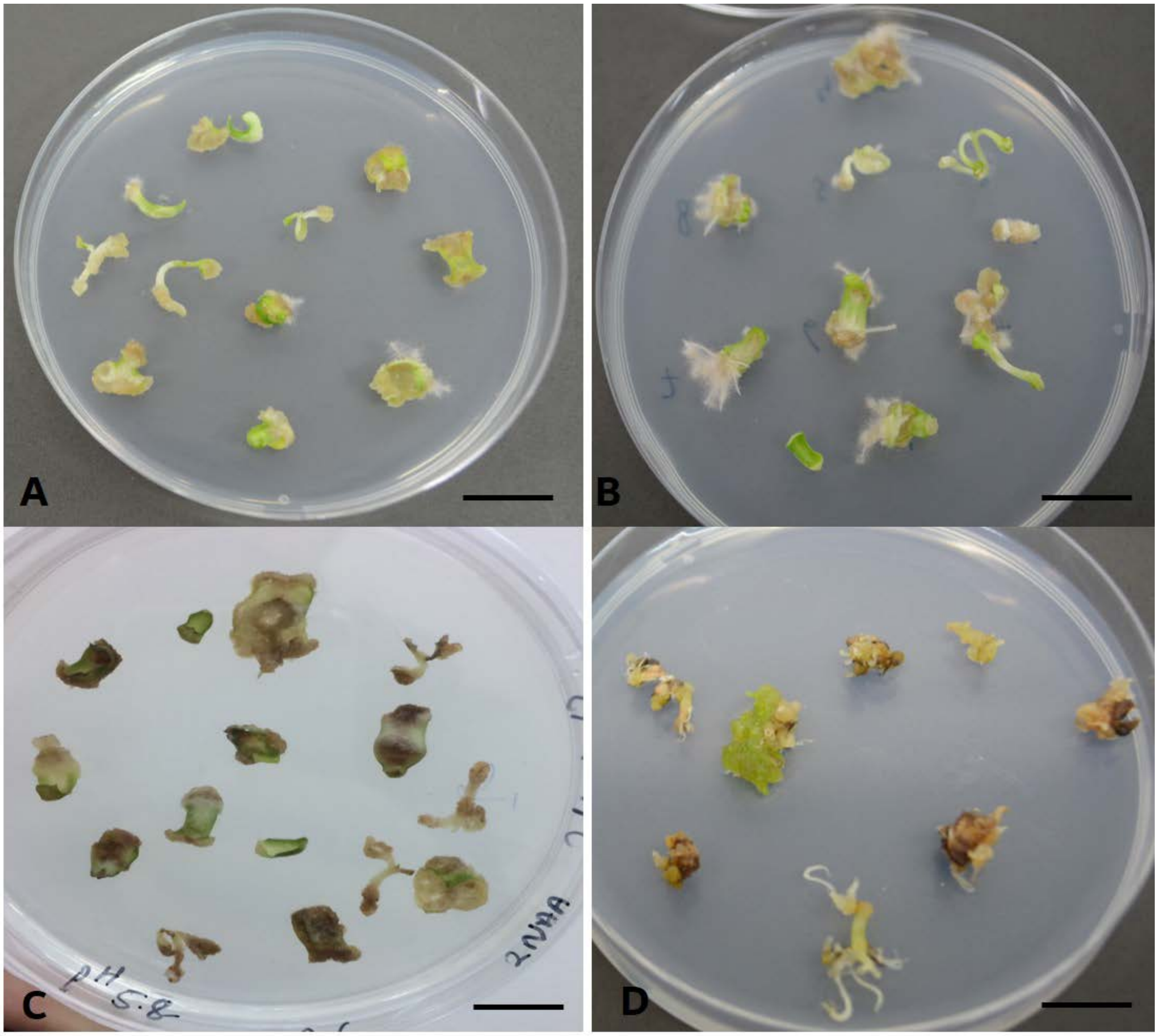

(A) Explants cultured on pH 4.0 + 2mg/L NAA in light conditions. (B) Explants cultured on pH 4.0 + 2mg/L NAA in dark conditions. (C) Explants cultured on pH 5.8 + 2mg/L NAA in light conditions. (D) Explants cultured pH 5.8 + 2mg/L NAA in dark conditions. Scale bars (A-D) 150 mm.
