# Supplementary material for: Efficient and reproducible somatic embryogenesis and micropropagation in tomato via novel structures - Rhizoid Tubers
Source: PLoS One. 2019 May 22;14(5):e0215929. doi: 10.1371/journal.pone.0215929 (PMC6530835; doi:10.1371/journal.pone.0215929)

Fig S4. Individual rhizoids excised and incubated on TDZ under light for *in vitro* shoot formation. Scale bar, 100mm.

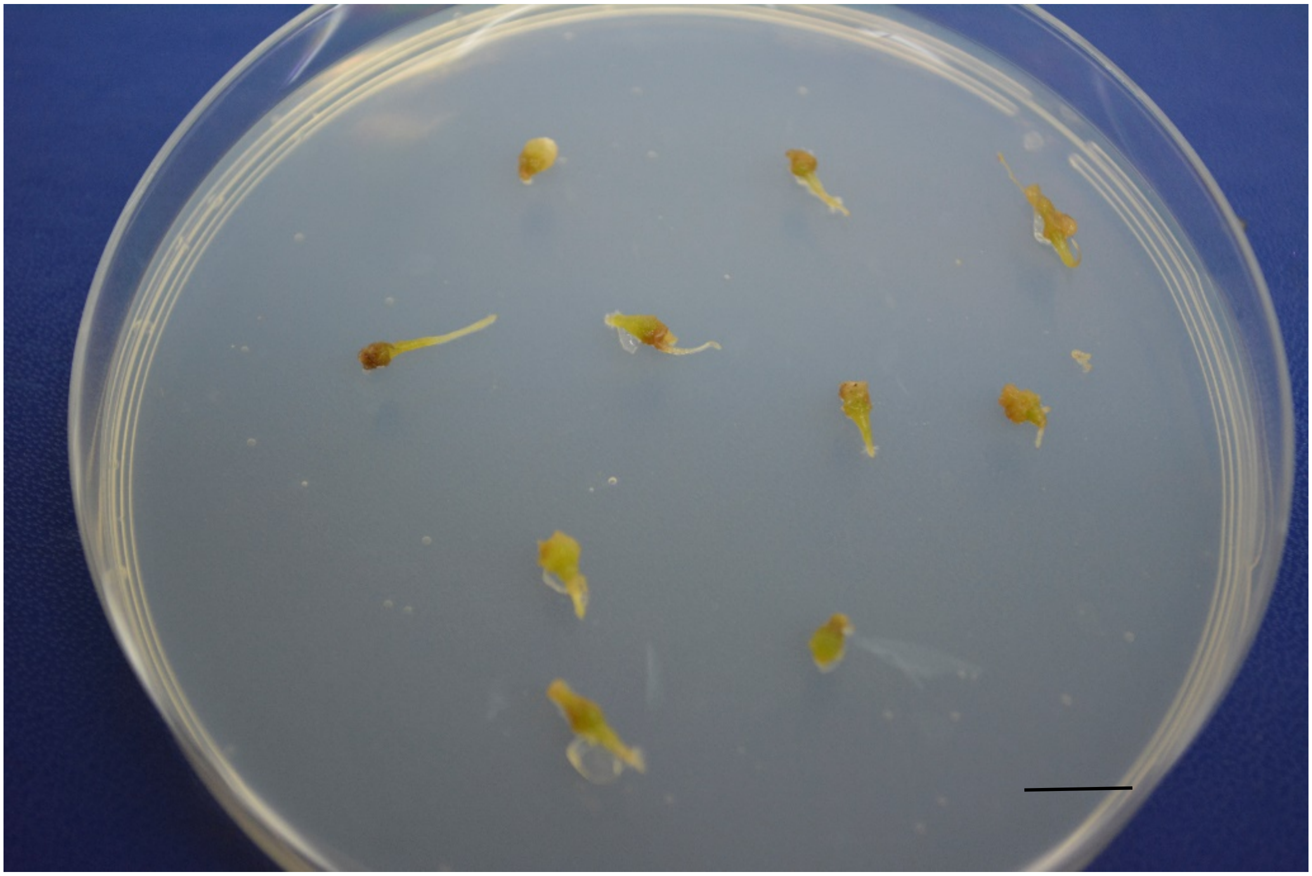

Supplement: S4 Fig — Scale bar, 100mm. (PDF) [file pone.0215929.s006.pdf]
