# Supplementary material for: Efficient and reproducible somatic embryogenesis and micropropagation in tomato via novel structures - Rhizoid Tubers
Source: PLoS One. 2019 May 22;14(5):e0215929. doi: 10.1371/journal.pone.0215929 (PMC6530835; doi:10.1371/journal.pone.0215929)
Supplement: S5 Fig — At an earlier stage, the cluster of rhizoid hair structure (A) was fixed and stained. (B) The cross-sectional image shows globular and bipolar embryos originating from sub-epidermal cells. Scale bar (A) 20mm. Scale bar (B) 50μm. (PDF) [file pone.0215929.s007.pdf]

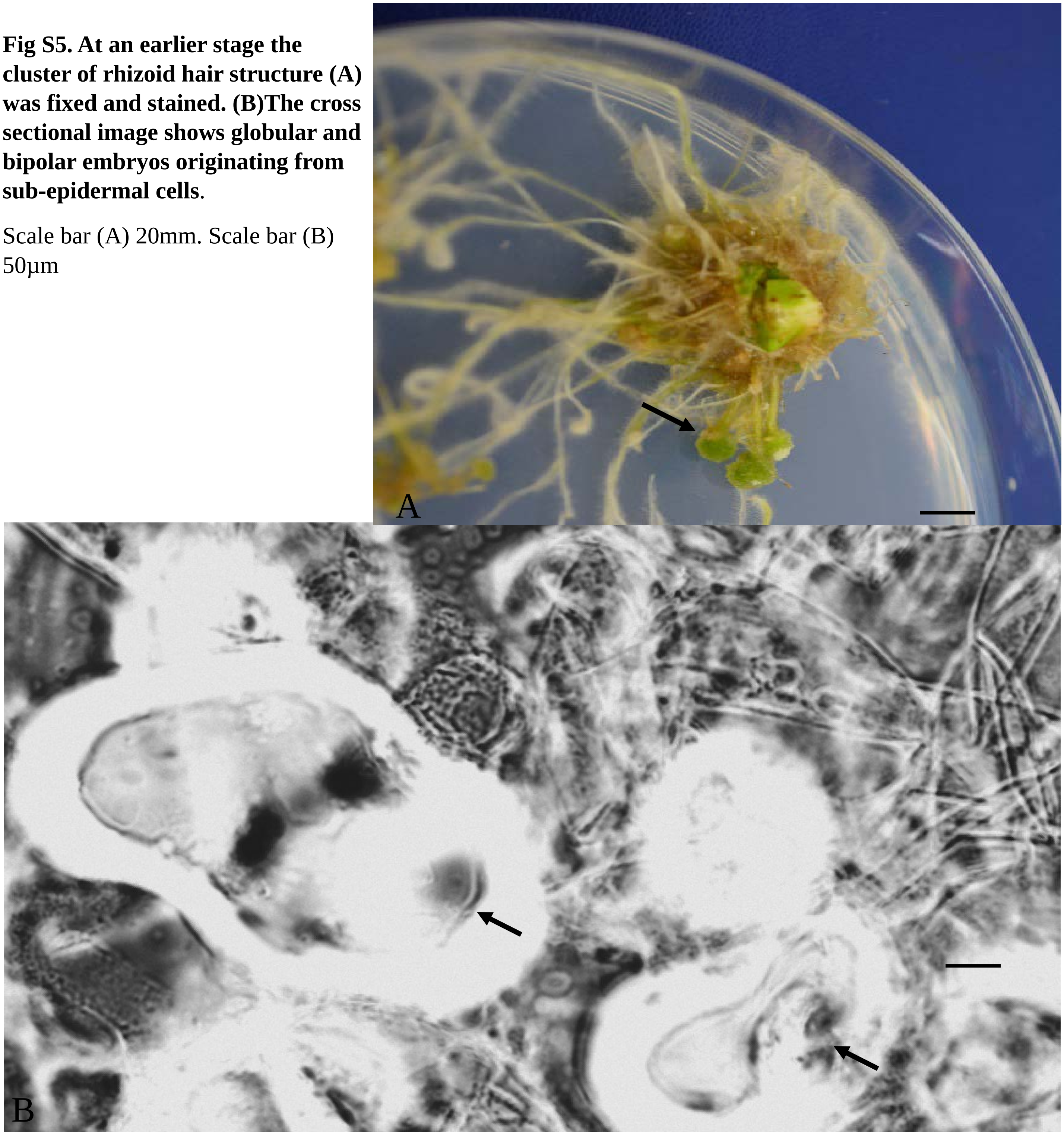

Fig S5. At an earlier stage the cluster of rhizoid hair structure (A) was fixed and stained. (B)The cross sectional image shows globular and bipolar embryos originating from sub-epidermal cells.

Scale bar (A) 20mm. Scale bar (B) 50µm
